# A Systematic Analysis of Cell Cycle Regulators in Yeast Reveals That Most Factors Act Independently of Cell Size to Control Initiation of Division

**DOI:** 10.1371/journal.pgen.1002590

**Published:** 2012-03-15

**Authors:** Scott A. Hoose, Jeremy A. Rawlings, Michelle M. Kelly, M. Camille Leitch, Qotaiba O. Ababneh, Juan P. Robles, David Taylor, Evelyn M. Hoover, Bethel Hailu, Kayla A. McEnery, S. Sabina Downing, Deepika Kaushal, Yi Chen, Alex Rife, Kirtan A. Brahmbhatt, Roger Smith, Michael Polymenis

**Affiliations:** 1 Department of Biochemistry and Biophysics, Texas A&M University, College Station, Texas, United States of America; 2 Department of Veterinary Pathobiology, Texas A&M University, College Station, Texas, United States of America; Fox Chase Cancer Center, United States of America

## Abstract

Upstream events that trigger initiation of cell division, at a point called START in yeast, determine the overall rates of cell proliferation. The identity and complete sequence of those events remain unknown. Previous studies relied mainly on cell size changes to identify systematically genes required for the timely completion of START. Here, we evaluated panels of non-essential single gene deletion strains for altered DNA content by flow cytometry. This analysis revealed that most gene deletions that altered cell cycle progression did not change cell size. Our results highlight a strong requirement for ribosomal biogenesis and protein synthesis for initiation of cell division. We also identified numerous factors that have not been previously implicated in cell cycle control mechanisms. We found that CBS, which catalyzes the synthesis of cystathionine from serine and homocysteine, advances START in two ways: by promoting cell growth, which requires CBS's catalytic activity, and by a separate function, which does not require CBS's catalytic activity. CBS defects cause disease in humans, and in animals CBS has vital, non-catalytic, unknown roles. Hence, our results may be relevant for human biology. Taken together, these findings significantly expand the range of factors required for the timely initiation of cell division. The systematic identification of non-essential regulators of cell division we describe will be a valuable resource for analysis of cell cycle progression in yeast and other organisms.

## Author Summary

What determines when cells begin a new round of cell division also dictates how fast cells multiply. Knowing which cellular pathways and how these pathways affect the machinery of cell division will allow modulations of cell proliferation. Baker's yeast is suited for genetic and biochemical studies of eukaryotic cell division. Previous studies relied mainly on cell size changes to identify systematically factors that control initiation of cell division. Here, we measured the DNA content of each non-essential single gene deletion strain to identify genes required for the correct timing of cell cycle transitions. Our comprehensive strategy revealed new pathways that control cell division. We expect that this study will be a valuable resource for numerous future analyses of mechanisms that control cell division in yeast and other organisms, including humans.

## Introduction

Understanding cell division requires knowing not only *how*, but also what determines *when* cells divide. Previous studies identified several components of the machinery that drives the cell cycle. However, it is not clear how cellular pathways impinge on the cell division machinery to initiate cell division. This is a critical gap in our understanding, since this process governs overall proliferation: once cells initiate their division, they are committed to completing it.

In proliferating cells, the G1 phase of any given cell cycle lasts from the end of the previous mitosis until the beginning of DNA synthesis. In unfavorable growth conditions, eukaryotic cells typically stay longer in G1, delaying initiation of DNA replication [1]–[7]. Subsequent cell cycle transitions, culminating with mitosis, are less sensitive to growth limitations, and their timing does not vary greatly, even if growth conditions worsen. Hence, differences in the length of the G1 phase account for most of the differences in total cell cycle, or generation times, between the same cells growing in different media, or among different cells of the same organism. Such fundamental observations support the notion that eukaryotic cells commit to a new round of cell division at some point in late G1 [3], [4], [8], [9]. Budding yeast cells also evaluate their “growth” in late G1 at a point called START, before DNA synthesis in S phase [1]. In favorable growth conditions, and in the absence of mating pheromones (for haploids), or meiotic inducers (for diploids), cells pass through START [1]. Passage through START and commitment to cell division precedes a large transcriptional program and additional events that lead to initiation of DNA replication [10]–[12].

The lack of a detailed view of upstream regulatory networks that govern the timing of START in the yeast *Saccharomyces cerevisiae* is surprising, given the rich history of the field. The classic *cdc* screen identified factors essential for START, such as Cdc28p [1], the main yeast cyclin-dependent kinase (Cdk). However, the *cdc* screen did not target nonessential regulators, such as the cyclin regulatory subunits of Cdc28p [13]. Other efforts relied on gene-specific suppression [14]–[18] or sensitivity to mating pheromones [19], [20]. By far, however, most approaches to identify regulators of START interrogated cell size. Almost half a century ago, a relationship between the size or mass of a cell and the timing of initiation of DNA replication was described from bacterial [21], to mammalian cells [22]. Indeed, a newborn budding yeast cell is smaller than its mother is, and it will not initiate cell division until it becomes bigger [1]. Thus, it appears that there is a critical size threshold for START completion in yeast. Based on this concept of a critical size, the question of “*when* do cells divide?” was reduced to “*what size* are cells when they divide?” Hence, several screens for regulators of START interrogated cell size [23]–[27]. In fact, systematic, genome-wide approaches to find genes required for the correct timing of START relied solely on cell size changes [23], [24].

Any gene deletion that alters the length of the G1 phase relative to the rest of the phases of the cell cycle will alter the DNA content profile. Thus, the DNA content of a population reports on the relative length of the G1 phase directly, discerning cells with unreplicated genome. In yeast, DNA content analyses measured the effects of gene over-expression on cell cycle progression [28], [29], or cycle arrest when essential genes were turned-off [30]. However, the yeast single-gene deletion collections have not been evaluated with this method.

To assess cell cycle progression more directly, we evaluated the yeast deletion collection of nonessential genes for altered DNA content, by flow cytometry. We found that most gene deletions that altered cell cycle progression did not change cell size. Our results suggest that evaluating the length of the G1 phase of the cell cycle, instead of cell size, provides a much more accurate view of the contribution of individual gene products to the timing of START and commitment to cell division. We also documented a strong requirement for ribosomal biogenesis for initiation of cell division, and identified numerous factors that have not been implicated previously in cell cycle control mechanisms. One such factor is the metabolic enzyme cystathionine-β-synthase (CBS; Cys4p in yeast). We discovered a novel, non-catalytic role of CBS, in accelerating START.

Taken together, the data we present here substantially expand the range of factors that affect initiation of cell division. We discuss the significance of our finding that most gene deletions that change the length of the G1 phase do not alter cell size, in the context of models that center on the role of cell size at START.

## Results

### Rationale and outline of the experimental design

We measured the DNA content during exponential growth in rich media (YPD-2% dextrose [31], see Methods), for several reasons: First, exponential growth in liquid media affords much greater reproducibility [32]. Second, for the haploid deletion strains, cell size measurements during the same growth conditions are available [23]. Third, fitness data during growth in the same rich media are available [33], providing another parameter for interpreting our findings.

**Figure 1:**
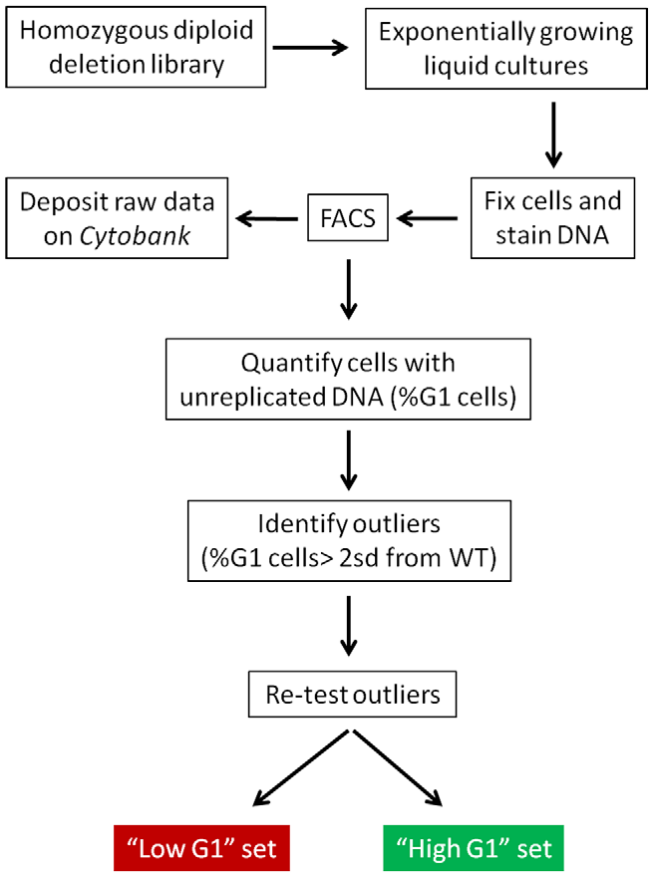
Schematic overview of our approach. For a detailed description of all the protocols we used, see Methods.

**Figure 2:**
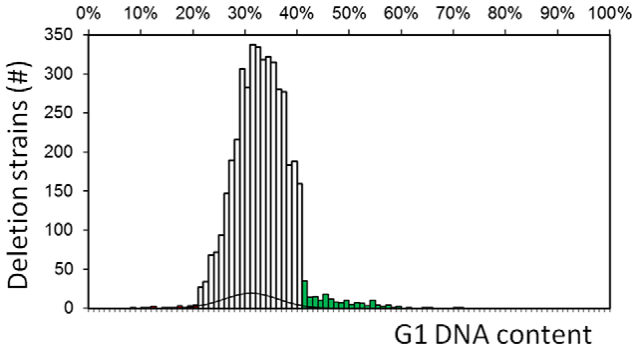
DNA content screen identifies genes required for normal cell cycle progression. Cumulative histogram displaying the percentage of cells in the G1 phase of the cell cycle (%G1), for homozygous diploid deletion strains. The bin width of the histogram is 1%, with each bin containing all the strains with values within the bin boundaries. The black line superimposed to this histogram is the normal distribution fit of the %G1 values of the reference wild type strain. Bins with values >2 sd from the mean of the wild type distribution are in red (“Low G1” group) and green (“High G1” group).

We used the homozygous diploid deletion panel to query the nonessential genes, to minimize the effects of aneuploidy found in a substantial portion of haploid deletion strains [34]. We evaluated strains individually (Figure 1). We quantified each sample in an automated manner, recording the percentage of cells with unreplicated genome (%G1, see Methods). We did not quantify complex profiles (e.g., due to cell separation defects, see Figure S1), and we excluded these strains from further analyses. At the beginning and end of most batches of strains, we measured the reference wild type strain (BY4743), which was cultured and processed along with the deletion strains. To identify strains with altered cell cycle, we compared the frequency distribution of the deletion strains against a normal distribution fit of the wild type (31.17%±5.20, n = 250) samples (Figure 2). Deletion strains that had a %G1 greater or less than two standard deviations of the wild-type distribution were considered to differ significantly from wild type, and we evaluated them further (see Methods).

### A large number of gene deletions affect the G1 phase of the cell cycle

From all strains analyzed successfully (n = 4,342; Dataset S1), 152 were in the “High G1” group, but only 16 were in the “Low G1” group. Hence, the majority of gene deletions that affect cell cycle progression lead to a G1 delay (Figure 2). We expect that additional gene deletions affect cell cycle progression, but were not included in the “High G1” or “Low G1” groups, for at least two reasons: experimental error; and imposition of restrictive cutoffs (>41.57%G1 for the “High G1” group and <20.77%G1 for the “Low G1” group). An example of the latter is *whi5Δ* cells, which lack an inhibitor of START [35], [36]. *whi5Δ* cells clearly had “Low G1” DNA content, with ∼25% of cells in G1 (compared to ∼31% for wild type cells), but that value was still within 2 sd of the WT mean (Figure 2). To examine the issue of false negatives in more detail, we determined the timing of START in two strains, which were close to our cutoffs, but not included in the candidate lists. Each of these strains lacked a protein kinase of unknown function: Kns1p [37] -*kns1Δ* cells had a 27% G1 score; or Tda1p [38] -*tda1Δ* cells had a 39% G1 score.

DNA content measurements from asynchronous cultures only reflect the *relative* duration of the G1 phase compared to the rest of the cell cycle phases. For example, a given deletion could increase the length of not only the G1 phase, but also subsequent phases. In that case, if the mitotic phases are disproportionately expanded compared to the G1 phase, that strain will display a “Low G1” DNA content, despite its lengthened G1 phase. To address this possibility, we obtained estimates of the *absolute* length of the G1 phase. The length of the G1 phase of a strain cultured in any given medium can be measured if one knows three parameters: i) The size of newborn cells (“birth” size). ii) The “critical size” these newborn daughter cells must attain to initiate cell division. iii) The rate (“growth rate”) at which they grow from their birth size to their critical size. Each of these variables is obtainable in yeast. From cell size distributions of log-phase cultures obtained with a channelyzer, daughter “birth” size was defined as the maximum size of the smallest 10% of cells on the left side of the cell size distribution of each strain. Wild type, *kns1Δ* and *tda1Δ* cells had indistinguishable cell size distributions (Figure S2A), and the same birth size (∼35 fl), in this medium (YPD-0.5% Dextrose). To obtain the “critical size” and “growth rate” of these strains, we examined highly synchronous, elutriated cultures [39]–[41]. As a function of time, we measured cell size and the percentage of budded cells (budding correlates with START completion). We found that there was no difference between wild type and *kns1Δ* cells (Figure S3). In contrast, *tda1Δ* cells delay START, not because they have altered critical size (Figure S3B), but because they reach that size slower than wild type cells do (Figure S3A). Hence, our cutoffs exclude some gene deletions with cell cycle effects, such as *whi5Δ* or *tda1Δ* cells. Therefore, despite the large number of gene deletions we identified to alter cell cycle progression significantly, we have likely underestimated that number.

### Most gene deletions that affect cell cycle progression do not alter cell size

**Figure 3:**
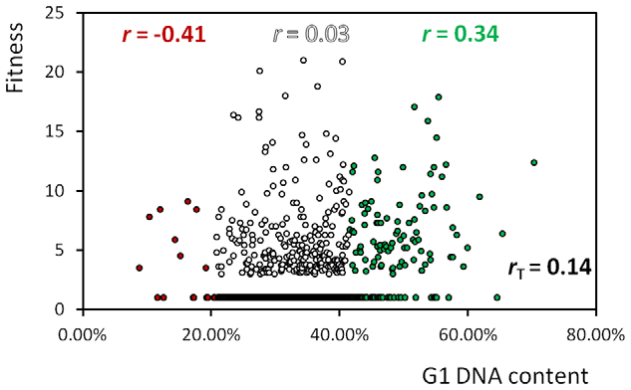
Decreased fitness correlates with altered cell cycle progression. The y-axis shows the fitness values of Giaever et al [33]. Higher values indicate reduced fitness. The cutoff for reduced fitness was about <85% of the wild type in that study [33]. Thus, strains with possible small reductions in fitness have been assigned a “WT-like” fitness score of 1. Giaever et al [33] evaluated fitness of the same strains we used, during growth in rich (YPD-2%Dextrose) liquid media, allowing for a direct comparison with our dataset. We used the non-parametric Spearman test to obtain the correlation (*r*) values we show. The correlation coefficient for all the strains (*r*_T_) is shown at the bottom right of the graph. We colored the r values for the sub-groups as in Figure 2. For every gene we included in this analysis, the values we used in this correlation are shown in Dataset S1.

We found that reduced fitness [33] correlates with altered cell cycle progression to some degree (Figure 3). Nevertheless, many gene deletions affect cell cycle progression, without affecting fitness. Cells that spend relatively more time in a particular cell cycle phase may not display reduced fitness because reciprocal, compensatory changes in the duration of other cell cycle phases may result in no net change in total generation time. Several known cell cycle mutants behave in this manner (e.g., *whi5* cells [23]).

**Figure 4:**
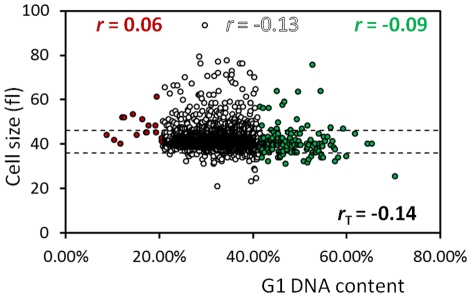
Cell size correlates poorly with DNA content. We plotted the %G1 (x-axis) from all the deletion strains we examined against the haploid median cell size (in fl, y-axis) data of Jorgensen et al [23]. The dashed lines indicate the cutoffs used in that study. We calculated and displayed the *r* values as in Figure 3. For every gene we included in this analysis, the values we used in this correlation are shown in Dataset S1.

We then compared %G1 values against cell size [23], [24]. We expected a strong negative correlation between cell size and the fraction of cells with unreplicated genome, since as cells advance in the cell cycle, the bigger the cells become. Remarkably, however, there was only a very weak, negative correlation between %G1 and cell size (r = −0.14, Figure 4 and Figure S4). Most of the deletion strains displaying a longer G1 (the “High G1” group) did not have altered cell size (Figure 4, strains between the dashed lines; and Figure S4). Conversely, many strains classified as size mutants [23], [24] did not have significantly altered DNA content (Figure 4, open circles outside the dashed lines, and Figure S4). These data show that changes in cell size are neither necessary nor sufficient for altered cell cycle progression. In the Discussion, we describe the implications of these results in the context of previous attempts to identify cell cycle regulators based on cell size changes.

Along with DNA content, we also analyzed the forward scatter (FSC) from the same flow cytometry experiments. FSC values often serve as a proxy for cell size, especially in animal model systems [42], [43]. An overall negative correlation between FSC values and %G1 was present (r = −0.26, Figure S5). However, we noticed some discrepancies. For example, in the “High G1” group %G1 correlated to some extent with FSC (*r* = −0.31), but much less with actual cell size (*r* = −0.09, Figure 4). We then correlated FSC values to cell size. Surprisingly, for the majority of strains, FSC values do not correlate well with published [23], [24] cell size values (Figure S6). These data suggest that inferring cell size phenotypes from FSC measurements may be problematic.

We next asked if there is a correspondence between genes that affect cell division when over-expressed, with genes required for normal cell cycle progression. We compared our data set to the genes identified in a systematic over-expression screen, which also relied on DNA content changes [28]. In only one case did over-expression of a non-essential gene have the reciprocal effect of its deletion (*NIP100*, encoding the large subunit of dynactin; Table S2). On the other hand, about half of the deletion strains with a low budding index [44] also had a high %G1 (Table S3). This is reasonable, since budding correlates with START completion [1].

### Deletion of genes involved in ribosomal biogenesis and protein synthesis delay START

The “Low G1” group is enriched for “cell cycle” gene ontologies (Table S4). We point out the *sic1Δ* strain, which was the 2^nd^-highest ranked strain of the group. Sic1p is a Cdk inhibitor of Clb/Cdk complexes, which is destroyed before cells initiate DNA replication [13]. Cells lacking Sic1p are not small size mutants [23], [24], and Sic1p was identified biochemically, as a Cdk-associated protein [45]. The “High G1” group is enriched for genes involved in “cytoplasmic translation” and “ribosome biogenesis” (Table S5). This is consistent with protein synthesis and ribosome biogenesis being required for the timely completion of START [2], [46]–[49].

In our analyses, we considered a high G1 DNA content and a lengthened G1 phase indicative of *delayed* START. We noticed that some of the genes involved in ribosome biogenesis and protein synthesis that we found with a “High G1” DNA content, were also classified by others as small size mutants with *accelerated* START [23], [50]. For example, *sfp1Δ* cells, which lack a transcription factor important for ribosome biogenesis [23], [51], [52], was the 2^nd^ highest-ranked gene deletion in our “High G1” group (see Figure S1 and Dataset S1). Yet, although the high G1 DNA content of *sfp1Δ* cells was noted [23], because of the small size of *sfp1Δ* cells, others concluded that START was accelerated in these cells [50].

**Figure 5:**
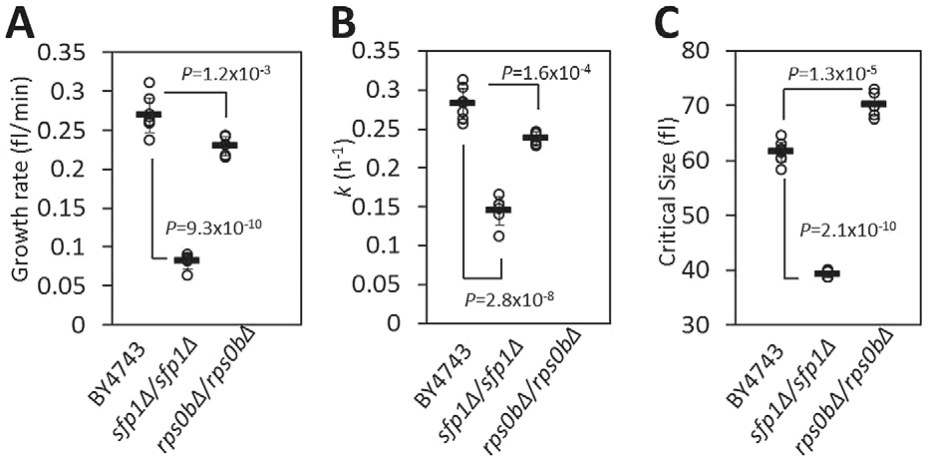
Deletion of genes involved in ribosome biogenesis delay START. A, Rate of cell size increase (shown as growth rate, in fl/min) for the indicated strains was measured from synchronous elutriated cultures, in YPD-2% Dextrose medium. The average value for each strain was calculated assuming linear growth and is shown with a horizontal bar (± sd). Where indicated, the *P* values shown were calculated from two-tailed *t* tests. The data used to calculate these values are shown in Figure S7A. B, The specific rate of cell size increase constant *k* (in h^−1^) was measured from the same elutriation experiments shown in A, assuming exponential growth. The data used to calculate these values are shown in Figure S7B. C, The critical cell size of the indicated strains (shown in fl), was measured from the same elutriation experiments shown in A and B (see also Figure S7C).

To resolve these discrepancies, we decided to examine transit through G1 and START completion in *sfp1Δ* cells. We did these experiments in YPD medium with 2% Dextrose, because Jorgensen et al used the same medium in a similar analysis of *sfp1Δ* cells [50]. Under these conditions, wild type cells have a “birth” size of 42.12±1.23 fl (n = 3) and a “critical” size of 61.53±0.64 fl (n = 8). We found that *sfp1Δ* cells had dramatically reduced “birth” (16.04±0.62 fl, n = 3, *P* = 6.9×10^−5^ based on a *t* test, see Figure S2B) and “critical” (39.23±0.53 fl, n = 6, *P* = 2.1×10^−10^, Figure 5C, Figure S7) sizes, and “growth rate” (Figure 5A and 5B, Figure S7). We calculated the “growth rate” differences between wild type and *sfp1Δ* cells in two different ways (see Methods), assuming that growth is exponential or linear. If growth is exponential, then *sfp1Δ* cells grow at ∼50% the rate of wild type cells (Figure 5B, Figure S7). If growth is linear, then *sfp1Δ* cells grow at ∼30% the rate of wild type cells (Figure 5A, Figure S7). For all other comparisons of “growth rates” between different strains that we present in this study, we obtain similar results, regardless of whether we plot size increases in an exponential or a linear manner, because the overall size of those strains is similar to wild type. However, given the strong cell size phenotype of *sfp1Δ* cells, and since exponential growth incorporates cell size differences (i.e., smaller cells grow slower than large cells), the growth rate decrease of *sfp1Δ* cells compared to wild type appears somewhat less if one assumes exponential increase in size. Nonetheless, regardless of whether growth is linear or exponential, it is clear that the G1 phase of *sfp1Δ* cells is substantially expanded (∼4-fold, see Methods for calculations). Cells lacking Sfp1p have a very long G1 because they are born very small, and they grow very slowly. Therefore, their small critical size notwithstanding, we conclude that START is severely delayed in *sfp1Δ* cells. We expand on this interpretation further in the Discussion.

To probe the connection between ribosomes and START further, we next evaluated *rps0bΔ* cells, another mutant with small size [23], lacking one of the Rps0 variants of the 40S ribosome particle. Cells lacking *RPS0B* have a high G1 DNA content (54%, see Dataset S1). We found that *rps0bΔ* cells have a reduced “birth” size (34.53±1.89 fl, n = 3, *P* = 0.007 based on a *t* test, see Figure S2B), an increased “critical” size (70.06±1.90 fl, Figure 5C, Figure S7), and a slow “growth” rate (Figure 5A and 5B, Figure S7). From these data, we conclude the following: i) since each of these changes alone would be sufficient to prolong G1, the combination of all three adequately explain the significant G1 delay of *rps0bΔ* cells, ii) “birth” size is not necessarily a predictor of “critical” size, and vice versa, since the two values can be highly discordant, as in *rps0bΔ* cells, and iii) DNA content measurements incorporate contributions of all these variables, including growth rate, successfully identifying the long G1 and delayed START of *rps0bΔ* cells.

**Figure 6:**
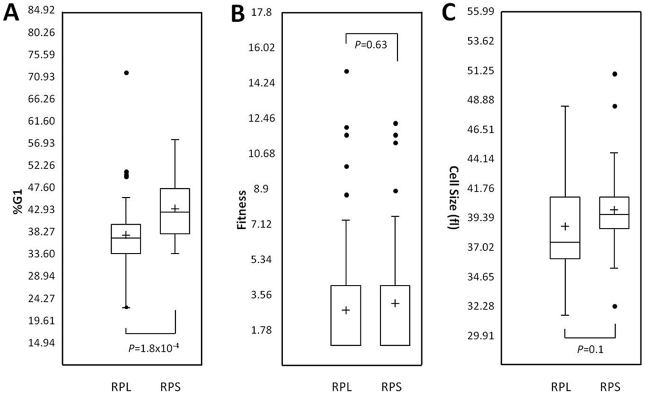
Phenotypes of ribosomal proteins. We grouped strains (n = 53) that lack ribosomal proteins of the 60S subunit (RPL), against strains (n = 43) that lack ribosomal proteins of the 40S subunit (RPS). We then compared the two groups based on the %G1 DNA content (this study; A), fitness (data from Giaever et al [33]; B), or haploid median cell size (data from Jorgensen et al [23]; C). The box plots were generated with Microsoft Excel. The box represents the middle 50% of the data range (from the 25th percentile to the 75th percentile). The band within the box is the median, while the cross shows the mean. The ends of the whiskers represent the lowest datum still within 1.5 of the interquartile range (IQR) of the lower quartile, and the highest datum still within 1.5 IQR of the upper quartile. Any data points not included within the whiskers are shown as outliers, displayed as filled circles. For the fitness data in B, the lower quartiles are not visible, because they are equal to 1 (i.e., most strains have fitness values similar to WT). The *P* values were calculated from *t* tests.

Next, we examined if there are any patterns in the requirement of ribosomal proteins for the timely completion of START. Intriguingly, although deletion of ribosomal protein subunits delayed START in general, the effect was much greater upon loss of 40S ribosomal proteins (RPSs), compared to the 60S subunits (RPLs; Figure 6A). In contrast, loss of RPSs or RPLs had similar effects on fitness (Figure 6B), or cell size (Figure 6C).

### Networks of genes affecting cell cycle progression

**Figure 7:**
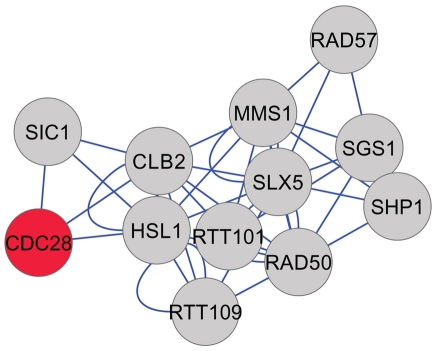
Network representation of the “Low G1” group. The interactions shown are from the gold-standard reference database BioGRID [54]. The network was constructed with Cytoscape [83], and displayed using an unbiased, force-generated layout. Only the factors that showed interactions (physical or functional) are included. We also included the essential gene *CDC28* (shown in red), encoding the major yeast Cdk.

**Figure 8:**
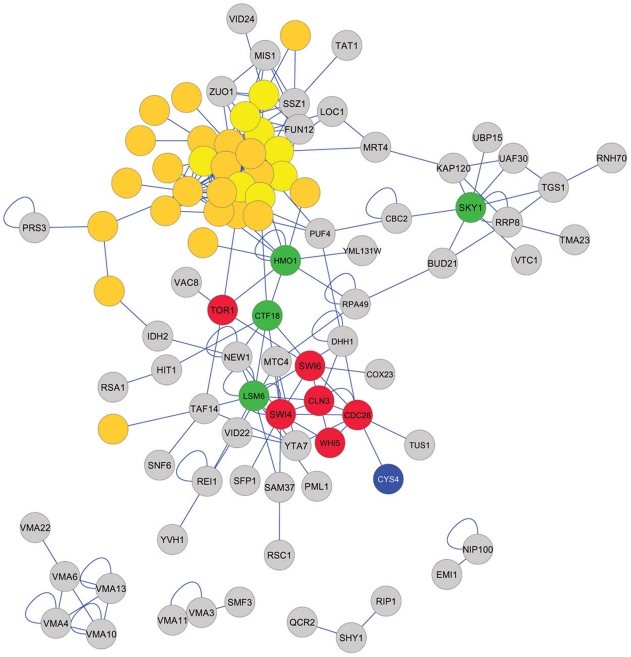
Interactions among the factors of the “High G1” group. The network of interactions was constructed and displayed as in Figure 7. We also included factors with known roles at START (shown in red), which were not identified in our study. Among the G1 cyclins, we only included Cln3p, which is responsible for initiating the positive feedback loop of the large G1/S transcriptional program [10]–[12]. The other G1 cyclins, Cln1p and Cln2p, are important for this feedback, once it is initiated by Cln3p, but they were not included in this network. 60S ribosomal proteins are in yellow, while 40S ribosomal proteins are in orange. The most highly connected factors among the ones we identified are in green, and Cys4p is in blue.

Factors with related biological functions show genetic interactions more often than expected by chance [53]. We queried the BioGRID database [54], for interactions among the genes we identified. Most of the factors of the “Low G1” group have multiple interactions with each other (Figure 7). In the “High G1” dataset, we also noted several highly connected factors (Figure 8), including the SR protein kinase Sky1p, similar to human SRPK1, which is involved in regulating proteins involved in mRNA metabolism. A group of genes in the “High G1” dataset that does not appear to interact with the rest of the group is composed of subunits of the vacuolar ATPase (Figure 8, bottom). Finally, we also noted an interaction between a metabolic enzyme, Cys4p, and the Cdk Cdc28p [55].

### A non-catalytic function of Cys4p promotes START

**Figure 9:**
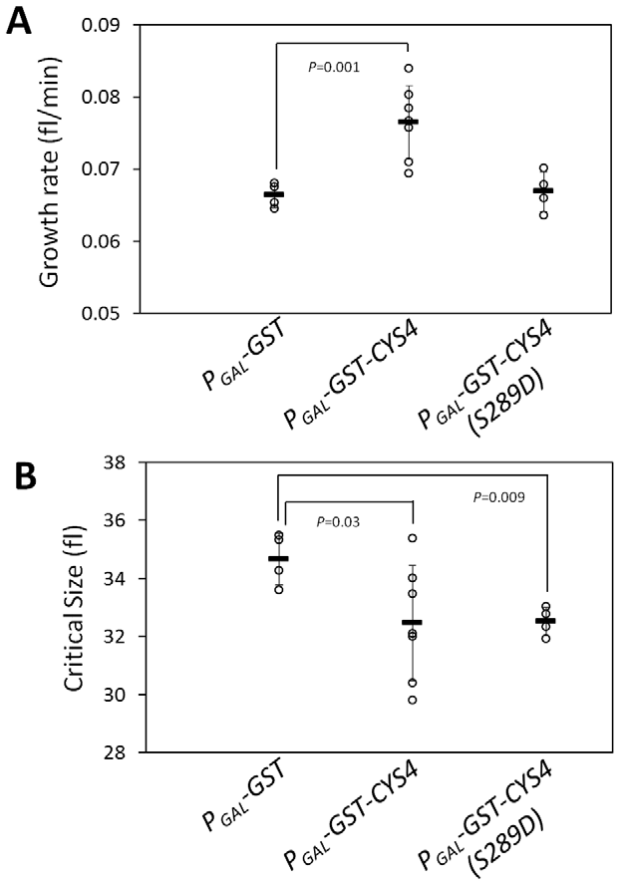
Cys4p advances START both by promoting cell growth and by a separate function, which does not require CBS's catalytic activity. A, Rate of cell size increase (shown as growth rate, in fl/min) for the indicated strains was measured assuming linear growth from synchronous elutriated cultures in media that contain galactose and induce expression of the *P_GAL_* alleles (see Methods). The average value for each strain is shown with a horizontal bar (± sd). Where indicated, the *P* values shown were calculated from two-tailed *t* tests. The data used to calculate the values shown in A and B are in Figure S8. B, The critical cell size of the indicated strains (shown in fl), was measured from the same elutriation experiments shown in A (see also Figure S9). The analogous experiments in non-inducing, glucose containing, medium are shown in Figure S9.

*CYS4* encodes the yeast CBS. We focused on Cys4p because we had previously shown that cells with a hypermorphic *CYS4* allele accelerate START [39]. Since the loss of Cys4p delays START (see Dataset S1), we queried the effects of Cys4p over-expression on START. To measure the timing of START, we examined highly synchronous, elutriated cultures. All strains cells had indistinguishable cell size distributions (Figure S2C) and the same birth size (∼14 fl, Figure S2C) in this medium (YPGal-3% Galactose). Consistent with Cys4p's metabolic role [39], we found that over-expression of Cys4p, but not of the catalytically inactive Cys4p-S289D variant [56], increased growth rate (Figure 9A). Over-expression of Cys4p also reduced the critical size for START (Figure 9B). Hence, wild type Cys4p accelerates START both by increasing growth rate, and by reducing critical size. Taking both of these variables into account, we conclude that over-expression of Cys4p shortens the length of the G1 phase by ∼30% (see Methods for calculations). Remarkably, over-expression of Cys4p-S289D also decreased critical size (Figure 9B, right). These results suggest that Cys4p promotes START in two ways: By promoting cell growth, which requires its catalytic activity; and by reducing critical size, which does not require Cys4p's catalytic activity.

**Figure 10:**
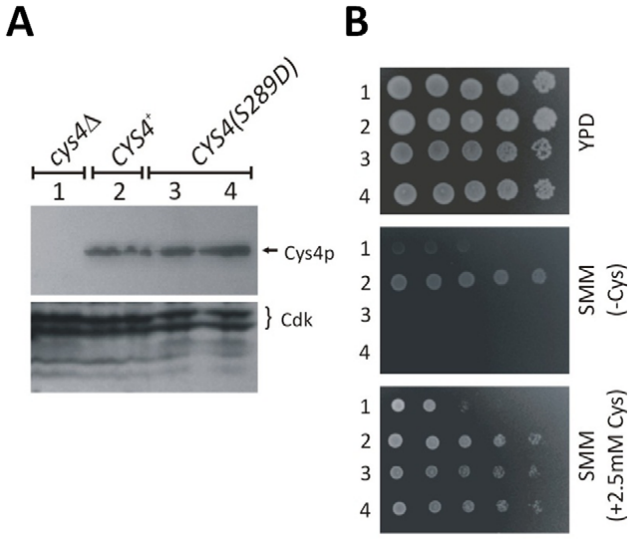
Cys4p has a vital, non-catalytic role in cell proliferation. A, Immunoblots showing the levels of Cys4p in the indicated strains, detected with an antibody against human CBS. We probed the same blot with an antibody against yeast Cdc28p, to indicate loading. B, Growth of the same strains on rich (YPD) and synthetic minimal media (SMM). We added cysteine (at 2.5 mM), to the SMM plate at the bottom. All strains were spotted on plates at 5-fold serial dilutions from liquid cultures, starting at ∼5,000 cells.

Yeast lacking *CYS4* can be viable if supplemented with cysteine [57]. In the standard S288c strain background we used here, *cys4Δ* cells proliferate slower than wild type (∼2 to 3-fold), even in rich media [33]. In humans, patients with CBS deficiency have high levels of homocysteine. These patients have brain, skeletal and vascular abnormalities [58]. There are more than 130 pathogenic CBS mutations, but not all of them affect the activity of CBS [59]. *Cbs* ^−^/^−^ mice have high levels of homocysteine (>200 µM) and die within weeks after birth [60]. In *Cbs* ^−^/^−^ mice, cells critical for the development of the cerebellum cannot proliferate [61]. Introducing human *CBS* alleles that encode inactive enzymes did not reduce the homocysteine levels of these mice, but these transgenes *did* rescue the neonatal lethality of *Cbs* ^−^/^−^ mice [62]. Thus, in animals, CBS must have essential, non-catalytic roles. Because of these observations, we asked if the catalytic role of Cys4p is separable from the proliferative defects associated with loss of Cys4p in yeast. We generated strains that express Cys4p-S289D at endogenous levels (Figure 10A, lanes 3 & 4). These strains are cysteine auxotrophs (Figure 10B, middle panel), consistent with their lack of Cys4p catalytic activity. However, when cysteine is present, they proliferate much better than strains that lack Cys4p altogether (Figure 10B, lower panel). These results are in remarkable agreement with the data in mice: Loss of CBS leads to proliferative and metabolic defects (homocysteinuria in mice, cysteine auxotrophy in yeast). In both organisms, inactive CBS does not suppress the metabolic defects, but it suppresses the proliferative defects.

## Discussion

Our results provide a comprehensive picture of the genetic requirements for the proper timing of initiation of cell division. The data we present raise several questions, and we discuss their implications and significance in the context of prevailing models of cell cycle control mechanisms.

### Why do most gene deletions that affect cell cycle progression lead to a G1 delay?

We think that this likely reflects the fact that cells commit to initiation of cell division in the G1 phase. It is reasonable to expect that extensive regulatory networks contribute to such a critical cellular transition, perhaps more so than for other cell cycle transitions. Interestingly, inactivation of the majority of essential genes also leads to a G1 arrest [30]. Furthermore, the strong requirement of protein synthesis for START completion [1], [2], [46]–[48], [63]–[65], and the large number of essential and non-essential genes involved in protein synthesis, also partially explains why most gene deletions that affect the cell cycle lead to a G1 delay.

### Is there a critical size threshold for initiation of cell division in yeast?

This question has been highly debated (see [66], [67] for related commentaries), especially when yeast is contrasted with animal model systems. Our study does *not* address this question. The debate about whether there is a critical threshold for initiation of cell division centers on whether cell size increases in a linear, or in an exponential fashion [66]–[69]. In several experiments, we monitored cell size increases as a function of time in synchronous cultures. However, our data points are of insufficient resolution to distinguish between an exponential vs. linear mode of growth (see Figure S7 and Methods). Note that this limitation does not in any way affect our conclusions about the relative rates of growth of different strains. In fact, when we compare strains with similar overall size distributions (see Figure S2A) the relative “growth rates” we obtain are the same, whether cells increase in size exponentially or not. Even in the case of strains with very different size distributions (e.g., wild type vs. *sfp1Δ* cells, see Figure S2B and Figure 5), the results are qualitatively similar, regardless of the pattern of growth. Nonetheless, in our study we have monitored and incorporated in our calculations the “critical size” at which cells initiate their division. From these experiments and similar others we published previously (see Figures S3, S7, S8, S9 and [39]–[41]), the “critical size” is a highly reproducible parameter. Hence, in accordance with numerous other reports, it is our opinion that any strain growing in a given medium has to reach a critical size characteristic of that strain and medium.

### Why do most gene deletions that affect cell cycle progression not affect cell size?

Our genome-wide data unequivocally show little correlation between %G1 and cell size (see Figure 4 and Figures S4, S5). Thus, although reaching a critical size threshold for initiation of cell division contributes to the timing of START, the most reasonable conclusion from our data is that genetic determinants of size control mechanisms are neither the sole nor the major factor determining the timing of initiation of cell division in dividing cells. This is a key finding of our study, which stands in marked contrast to previous approaches that used cell size alterations as a means to identify START regulators [23]. In our opinion, monitoring the length of the G1 phase reflects the timing of START far more accurately than monitoring cell size. We expand more on this issue next, when we discuss the role of ribosome biogenesis and the behavior of wild type cells in different nutrients.

### Does ribosome biogenesis promote or delay START in yeast?

The behavior of strains lacking genes involved in ribosome biogenesis and protein synthesis exemplifies the different interpretations about the timing of START, depending on whether the focus is on the length of G1 (this study), or on cell size [23], [50]. We will discuss the phenotypes of *sfp1Δ* cells, because we examined them (see Figure 5) with the same methods and under the same conditions as in previous studies by Jorgensen et al [50]. The parameters we obtained are in complete agreement with those of Jorgensen et al [50]: *sfp1Δ* cells divide at a greatly reduced cell size, grow much more slowly than wild type cells, and they are also born very small. Jorgensen et al focused on their small critical size and concluded that START was accelerated in *sfp1Δ* cells and other strains lacking genes involved in ribosome biogenesis [50]. Instead, we took into account not only their small critical size, but also their extremely slow growth rate and small birth size (see Figure 5 and Figures S2, S7). We conclude that START must be severely delayed in *sfp1Δ* cells, because these cells have such an expanded G1. If one focuses only on the small critical size of *sfp1Δ* cells, it may seem that START is accelerated. However, we think it is more accurate to describe these cells simply as small and severely growth-impaired. Loss of Sfp1p delays START to such an extent that during the time *sfp1Δ* cells spend in G1, their wild type counterparts would have initiated several new rounds of cell division.

Not all gene deletions that affect ribosome biogenesis prolong G1 and those who do may differ quantitatively and qualitatively in their impact (Figure 5, Figure 6). Overall, however, there is a prolongation of the G1 phase in many ribosome biogenesis mutants (see Dataset S1). Because of their lengthened G1, we conclude that START is delayed in strains lacking non-essential ribosomal components or factors that regulate protein synthesis. This interpretation is consistent with the terminal G1 arrest of essential genes involved in the same processes [30], and with the strong delay of START upon inactivation of rRNA processing in yeast [48]. For these reasons, we conclude that gene deletions that impair ribosome biogenesis delay START, and that in dividing wild type yeast cells, ribosome biogenesis *promotes* START. This conclusion also agrees with extensive evidence from animal cells that increased ribosomal biogenesis (by Myc and other oncogenes) promotes initiation of cell division [70]–[73].

### Does the length of the G1 phase accurately reflect the timing of START?

Obviously, completion of START and commitment to a new round of cell division precedes the actual end of the G1 phase, when cells initiate DNA replication [1], [11]. Mutants in processes that molecularly link START with DNA replication (e.g., *cdc34* cells [74]), may complete START, but they are unable to initiate DNA replication. These rare exceptions notwithstanding, we see no compelling reason that invalidates using the length of the G1 phase as an accurate metric of the timing of START. This is supported further by the behavior of dividing wild type cells in different growth conditions. Poor growth conditions greatly prolong G1, whereas the time required to transit the remaining cell cycle phases is unaffected [3]. In steady-state chemostat cultures, where growth rate can be altered independently of nutrient composition, the lower the growth rate is, the longer the cells stay in G1, delaying START completion [40], [75], while cell size remains largely unaffected [75], [76]. Nutrients also affect the critical size threshold. Cells dividing in poor carbon sources typically are small, but they also have a slow growth rate and a long G1 [77], resembling ribosome biogenesis mutants with a delayed START.

We would like to clarify that, in all of the above examples we discussed, we considered continuously dividing populations, without media changes. In a nutritional up-shift, from poor to rich media, G1 is transiently prolonged, probably until cells reach the new larger “critical size” characteristic of the rich medium [78]. During this short temporal window, in the first cell cycle as cells transit from the poor medium to the new richer one, genetic control of the “critical size” threshold likely prolongs G1 and delays START by increasing the critical size threshold [50], [79]. In subsequent cell cycles however, despite the larger “critical size” cells have to attain in that richer medium, the cells are born larger and grow faster, resulting in a short G1 and accelerated START.

What could be the benefit of the small critical size observed in poor nutrients? It has been argued that the plasticity of critical size thresholds may allow yeast cells to “best compete for limited and fluctuating resources” [79]. This is reasonable, if one keeps in mind the two competing objectives of all proliferating cells: i) Ensure that growth requirements are met before initiating a new round of cell division; ii) At the same time, exploit all the available nutrients to divide as quickly and as many times as possible. Perhaps, with their smaller birth size and slower growth rate, which lengthen G1, cells in poor nutrients satisfy the first objective. Then, as they progress in G1, cells have to reach a smaller critical size, alleviating a little bit the overall delay in initiating a new round of cell division in poor nutrients.

### Implications for our understanding of genetic networks that control initiation of cell division

Overall, our results increase the number of gene deletions that delay G1, as listed currently in the *Saccharomyces Genome Database*, by more than 3-fold. Even if one excludes genes involved in ribosome biogenesis, we still uncovered >100 genes required for the timely initiation of cell division (see Dataset S1). Most of the genes we identified do not affect cell size. As a result, these genes were not identified in previous attempts to find regulators of START. Hence, our findings greatly expand and reshape our view of START. We followed up one such gene we identified in this study, Cys4p (CBS). CBS is a key metabolic enzyme, associated with disease in humans, with conserved functions between yeast and humans. Indeed, human CBS complements yeast lacking Cys4p [80]. Hence, the role of CBS in cell division we described in yeast may be significant for human biology. The systematic identification of non-essential regulators of START we described here will be the basis for further insight into the control of cell division in yeast and other organisms. It enables future studies to define how many pathways affect START, which factors operate within each pathway, and the extent of interactions between pathways.

## Methods

### Yeast protocols

*S. cerevisiae* strains used in this study are listed in Table S1. Unless noted otherwise, we used standard yeast methods [31]. To construct the *P_GAL_-GST-CYS4* strain (Figure 9 and Figures S8, S9), we started from a commercially available plasmid containing a *P_GAL_-GST-CYS4* allele (Open Biosystems, cat#: YSC3869-95169400). However, this plasmid contained a frameshift mutation at nucleotide position 856 of the *CYS4* ORF, which we corrected. We then removed a BsrGI-SalI fragment, re-ligated the plasmid, and digested it with StuI. Finally, we integrated this linearized plasmid derivative containing the *P_GAL_-GST-CYS4* allele at the *URA3* locus of W303-k699 (see Table S1). We sequenced a similar plasmid supposed to carry a *P_GAL_-GST-KIP3* allele (YSC3869-9518649), but we found that it only drives expression of GST, due to downstream mutations. We used this plasmid to construct the negative control *P_GAL_-GST* strain (Figure 9 and Figures S8, S9), as we described above. From the *P_GAL_-GST-CYS4* plasmid we generated the *P_GAL_-CYS4(S289D)* derivative, as follows: We used the *P_GAL_-GST-CYS4* plasmid as a template in a PCR reaction with a forward primer encoding the S289D substitution, and a reverse primer complementary to sequences downstream of the *CYS4* ORF. The PCR fragment was then used to gap-repair the *P_GAL_-GST-CYS4* plasmid, which was previously digested with BstEII. The resulting *P_GAL_-CYS4(S289D)* plasmid was then used in the same way as above, to construct the *P_GAL_-CYS4(S289D)* strain (Figure 9 and Figures S8, 9). All plasmids were sequenced and the resulting strains were verified for expression of the proteins of interest.

The *CYS4-13MYC* strain (Figure 10) was made with a single-step PCR replacement [39]. To make the *CYS4(S289D)-13MYC* strain (Figure 10), we used genomic DNA of the *CYS4-13MYC* strain as a template in a PCR reaction with a forward primer encoding the S289D substitution, and a reverse primer complementary to sequences downstream of the *CYS4* ORF.

For DNA content measurements, strains were cultured standing at 30°C in YPD (1% yeast extract, 2% peptone, 2% dextrose). Overnight cultures were diluted 1∶500 into 1 ml fresh medium, cultured for 4–5 hrs, collected by centrifugation and fixed in 70% ethanol. To obtain size distributions from asynchronous cultures, overnight cultures of the strain and medium of interest were diluted 1∶500 in fresh medium, and allowed to proliferate for 5–6 h, before we analyzed them. For synchronous cell cycle analyses [39], strains were cultured and elutriated in YPD medium containing 0.5% dextrose (Figures S3, S9), 2.0% dextrose (Figure 5, Figure S7), or YPGal (1% yeast extract, 2% peptone, 3% galactose; Figure 9, Figure S8), as indicated.

### Cell size determinations

Cell size was measured with a Beckman Z2 Channelyzer. For each sample we analyzed, we obtained size distributions from two different dilutions of cells. The average of the geometric mean of each size distribution was recorded. We used the Accucomp Beckman software package to obtain the statistics of each size distribution.

### Measurements of critical size and growth rate from elutriated cultures

For isolation of early G1 daughter cells, cultures were grown in the medium indicated in each case at 30°C to a density of ∼1–5×10^7^ cells/ml, then fractionated with a Beckman JE-5.0 elutriator as described previously [41]. Early fractions containing predominantly (>95%) small unbudded cells were collected by centrifugation, resuspended in the medium indicated in each case and incubated at 30°C. Every 20 min we monitored the percentage of budded cells and cell size. The “critical size” is the size at which 50% of the cells have budded in these experiments, and it was calculated as we described elsewhere [41]. We calculated the rate of size increase, “growth rate” (in fl/min), assuming linear growth, as we described previously [41]. To calculate “growth rate” assuming exponential growth, we plotted the natural log (ln) of cell size as a function of time (in h), see Figure S7B. We fit the data to a straight line using the regression function in Microsoft Excel. From the slope of the line, we obtained the specific rate of cell size increase constant (*k*, in h^−1^). The average of all experiments for each strain was then calculated, along with the associated standard deviation. Since sometimes it takes the cells longer to recover from the elutriation, in our growth rate calculations we exclude this “lag” phase. We derived growth rate data only from the linear portion of each experiment.

Estimates of the length of G1 were calculated as follows: Assuming linear growth, G1_(min)_ = (“Critical Size”-“Birth Size”)/”Growth Rate”. Assuming exponential growth, G1_(h)_ = ln(“Critical Size”/”Birth Size”)/*k*.

### Staining for DNA content analyses

Fixed cells were stored at 4°C overnight to 14 days. Cells were collected by centrifugation and stained overnight in 1 ml staining solution containing 50 mM sodium citrate pH 7.0, 0.25 mg/ml RNaseA, and 500 nM SYTOX Green (Molecular Probes, OR). Samples were stored at 4°C overnight in opaque containers. Cell suspensions were sonicated briefly at the fixing and staining steps and immediately before flow cytometry.

### Flow cytometry data acquisition, deposition, and analysis

Stained cells were analyzed on a FACSCalibur (Becton Dickinson Immunocytometry Systems, CA) flow cytometer, using CellQuest (version 3.3; Becton Dickinson Immunocytometry Systems) acquisition software. Sytox Green fluorescence was collected through a 515/30-nm bandpass filter, and list mode data were acquired for 10,000 cells defined by a dot plot of FSC versus SSC. Prior to each experiment, standard beads (Cyto-Cal Multifluor Intensity Beads, Thermo Scientific, CA) were used to calibrate the flow cytometer, and photomultiplier tube voltages were adjusted to place the highest intensity bead in the same channel (+/−3). FACS files were archived at *Cytobank* [81]. Automated quantification of the DNA content histograms was done with FlowJo 7.5 software. To exclude particulate non-yeast events, which had both very low forward scatter (FSC) and low fluorescence (FL1/2-A), asymmetrical gates were fitted with the autogating tool. Gates were centered near FSC ∼100 and FL1/2-A ∼300 and contained all events of sufficient contiguity as defined by the default autogating parameters, on average ∼91% of total. From the gated populations, we determined the mean and standard deviation of the FSC parameter. Cell cycle phase subpopulations were computed from the gated population using the Dean-Jett-Fox model without constraints. %G1 was defined as the area of the G1 model peak, divided by the combined areas of the G1 and G2/M peaks. Because the %G1 results represent a continuum, it was necessary to impose cutoffs in order to exclude model fits that did not accurately represent experimental data. This was assessed primarily by root mean square (RMS) error, which averaged 11.68 (+/− a standard deviation of 2.80) across all included experiments. For this reason, we did not analyze experiments that yielded an initial model fit RMS >25, %G1<5%, or %G1>95% (since extremes in %G1 were often indicative of poor fit), except in a few cases where the model fit was visually inspected and/or manually constrained. Experiments for which the %G1 fell outside two standard deviations of the wild-type distribution were repeated additional times. Experimental data and correlations are provided in the searchable spreadsheet available as Dataset S1. Raw data files can be freely accessed at Cytobank (www.cytobank.org) and are found in the public experiments “Yeast DNA Content Project – DELETION – INCLUDED”, and “Yeast DNA Content Project – DELETION – EXCLUDED”.

### Statistical analysis

Non-parametric Spearman tests were done with the Analyze-it software package. In all other cases, statistical calculations were done with Microsoft Excel. Where indicated, *t* tests were 2-tailed, assuming unequal variance between data sets.

### Yeast protein extracts

Protein extracts for immunoblots were made with the NaOH extraction method [82].

### Antibodies

For detection of proteins of interest on immunoblots we used an anti-PSTAIR antibody to detect Cdk (Figure 10A; Abcam, Cat#: ab10345) and an anti-hCBS polyclonal antibody to detect human and yeast CBS proteins (Figure 10A; SantaCruz, Cat#:46830). Secondary antibodies were from Pierce. All antibodies were used at the dilutions recommended by the manufacturers.

## Supporting Information

Dataset S1 Searchable spreadsheet of all the primary data, arranged in different worksheets. In the worksheet entitled “Data and Correlation”, we list the experimental data we obtained, representing mean average values for individual deletions, organized by plate ID. Data from our study are correlated to growth and cell size data from the indicated studies, and descriptions from the Saccharomyces Genome Database (SGD; http://www.yeastgenome.org/). In the worksheet entitled “INCLUDED Experiments”, we list all our flow cytometry individual experiments that were included in the final analysis. Raw .fcs files can be accessed at Cytobank (www.cytobank.org). Public experiment name: “Yeast DNA Content Project – DELETION – INCLUDED.” In the worksheet entitled “EXCLUDED Experiments”, we list individual experiments that were excluded from the final analysis for various reasons, but which may represent valid flow cytometry profiles. Cytobank public experiment name: “Yeast DNA Content Project – DELETION – EXCLUDED.” Finally, in the worksheet entitled “Explanation,” we provide further detailed descriptions of each parameter listed in the previous worksheets.
(XLS)

Figure S1 Representative DNA content histograms. Three independent experiments of the indicated strains are shown in each case. Fluorescence is plotted on the x-axis, while the number of cells analyzed is on the y-axis. BY4743 is the wild type, diploid reference strain. *sfp1Δ/sfp1Δ*, or *rad57Δ/rad57Δ*, strains were from the “high G1”, or “Low G1” sets, respectively. *clb5Δ/clb5Δ*, or *elm1Δ/elm1Δ*, strains have known roles during DNA replication, or cytokinesis and cell separation, respectively, giving rise to complex DNA content histograms that were not quantified.
(JPG)

Figure S2 Cell size distributions of asynchronous cultures. The cell size of the indicated cell populations was measured using a channelyzer (see Methods). Cell numbers are plotted on the y-axis and the x-axis indicates size (in fl). A, Size distributions of wild type (BY4743), *kns1Δ/kns1Δ* and *tda1Δ/tda1Δ* cells, cultured in YPD (0.5% Dextrose) medium. B, Size distributions of wild type (BY4743), *sfp1Δ/sfp1Δ* and *rps0bΔ/rps0bΔ* cells, cultured in YPD (2% Dextrose) medium. C, Size distributions of wild type *pGAL-GST*, *pGAL-CYS4*, *pGAL-CYS4(S289D)* cells, cultured in YPGal (3% Galactose) medium.
(JPG)

Figure S3 Evaluating false negatives. A, Rate of cell size increase (shown as growth rate, in fl/min) for the indicated strains was measured from synchronous cultures, in rich (YPD-0.5% Dextrose) medium, assuming linear growth. The average value for each strain is shown with a horizontal bar (± sd). B, The critical cell size of the indicated strains (in fl), was measured from the same experiments shown in A. C, Graphs from which we determined the growth rates shown in A. D, Graphs from which we determined the percent of budded cells as a function of cell size, from the same elutriation experiments. The data points shown were from the linear portion of each experiment, when the percentage of budded cells began to increase, and used to determine the critical size for division we show in B.
(JPG)

Figure S4 Cell cycle progression correlates weakly with cell size data from stationary phase growth. We plotted the %G1 (x-axis) from all the deletion strains we examined against the diploid median cell size (in fl, y-axis) data of Zhang et al (24), in stationary phase after growth on solid media. We calculated and displayed the *r* value as in Figure 3. For every gene we included in this analysis, the values we used in this correlation are shown in Dataset S1.
(JPG)

Figure S5 Correlation between DNA content and FSC values. The %G1 is shown on the x-axis, and the forward angle scattering (FSC) values on the y-axis, from all the deletion strains we examined by flow cytometry. We calculated and displayed the *r* values as in Figure 3. For every gene we included in this analysis, the values we used in this correlation are shown in Dataset S1.
(JPG)

Figure S6 Correlation between FSC and cell size values. We plotted the FSC values (y-axis) from all the deletion strains we examined against the median cell size (in fl, x-axis) data of Jorgensen et al (23) (A), or Zhang et al (24) (B). We calculated and displayed the *r* values as in Figure 3. For every gene we included in this analysis, the values we used in this correlation are shown in Dataset S1.
(JPG)

Figure S7 Determining the timing of START in mutants that affect ribosome biogenesis. A, Graphs from which we determined the rate of cell size increase shown in Figure 5A, assuming linear growth. Our measurements were from synchronous cultures, in rich (YPD-2% Dextrose) medium. B, Graphs from which we determined the specific rate of cell size increase constant *k*, shown in Figure 5B, from the same elutriation experiments shown in A. In this case, we plotted the natural log of the cells size (y-axis), against time (shown in hours, x-axis). C, Graphs of the fraction of budded cells (y-axis) as a function of cell size (in fl, x-axis), from the same elutriation experiments. The data points shown were from the linear portion of each experiment, when the percentage of budded cells began to increase, and used to determine the critical size for division we show in Figure 5C.
(JPG)

Figure S8 Cell cycle progression of synchronous cultures of *P_GAL_* haploid strains, in galactose-containing media. The full data set used to calculate the values shown in Figure 9A and 9B, are shown on the left, and right panels, respectively. Elutriations were done in media that contain galactose and induce expression of the *P_GAL_* alleles (see Methods).
(JPG)

Figure S9 Cell cycle progression of synchronous cultures of *P_GAL_* haploid strains, in repressive, glucose-containing media. A, The rate of cell size increase (shown as growth rate, in fl/min) for the indicated strains was measured from synchronous elutriated cultures assuming linear growth, as in Figure 9, in media that contain glucose (YPD-0.5% Dextrose) and repress expression of the *P_GAL_* alleles. The average value for each strain is shown with a horizontal bar (± sd). B, The critical cell size of the indicated strains (shown in fl), was measured from the same elutriation experiments shown in A. The rate of cell size increase for each elutriation experiment of the indicated strains is shown on the left panels. C, D, The full data set used to calculate the values shown in A, and B, respectively.
(JPG)

Table S1 *S. cerevisiae* strains used in this study.
(DOCX)

Table S2 Correspondence between genes that affect cell division when over-expressed, with genes required for normal cell cycle progression.
(DOCX)

Table S3 Correspondence between gene deletions that affect the budding index and the DNA content.
(DOCX)

Table S4 Gene Ontology enrichment of the “Low G1” group.
(DOCX)

Table S5 Gene Ontology enrichment of the “High G1” group.
(DOCX)
