# Supplementary figures and images for: A Systematic Analysis of Cell Cycle Regulators in Yeast Reveals That Most Factors Act Independently of Cell Size to Control Initiation of Division
Source: PLoS Genet. 2012 Mar 15;8(3):e1002590. doi: 10.1371/journal.pgen.1002590 (PMC3305459; doi:10.1371/journal.pgen.1002590)

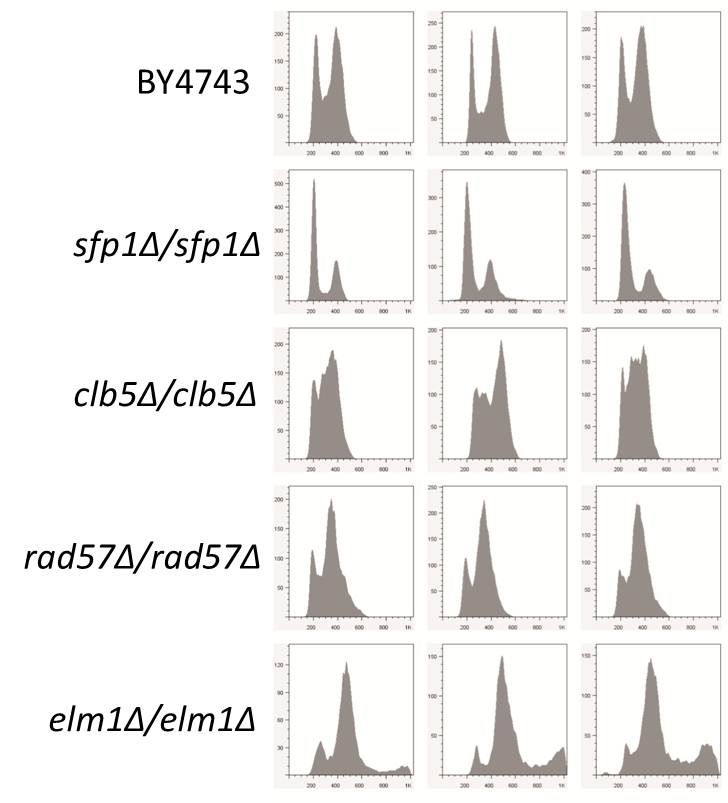

Supplement: Figure S1 — Representative DNA content histograms. Three independent experiments of the indicated strains are shown in each case. Fluorescence is plotted on the x-axis, while the number of cells analyzed is on the y-axis. BY4743 is the wild type, diploid reference strain. sfp1Δ/sfp1Δ, or rad57Δ/rad57Δ, strains were from the “high G1”, or “Low G1” sets, respectively. clb5Δ/clb5Δ, or elm1Δ/elm1Δ, strains have known roles during DNA replication, or cytokinesis and cell separation, respectively, giving rise to complex DNA content histograms that were not quantified. (JPG) [file pgen.1002590.s002.jpg]

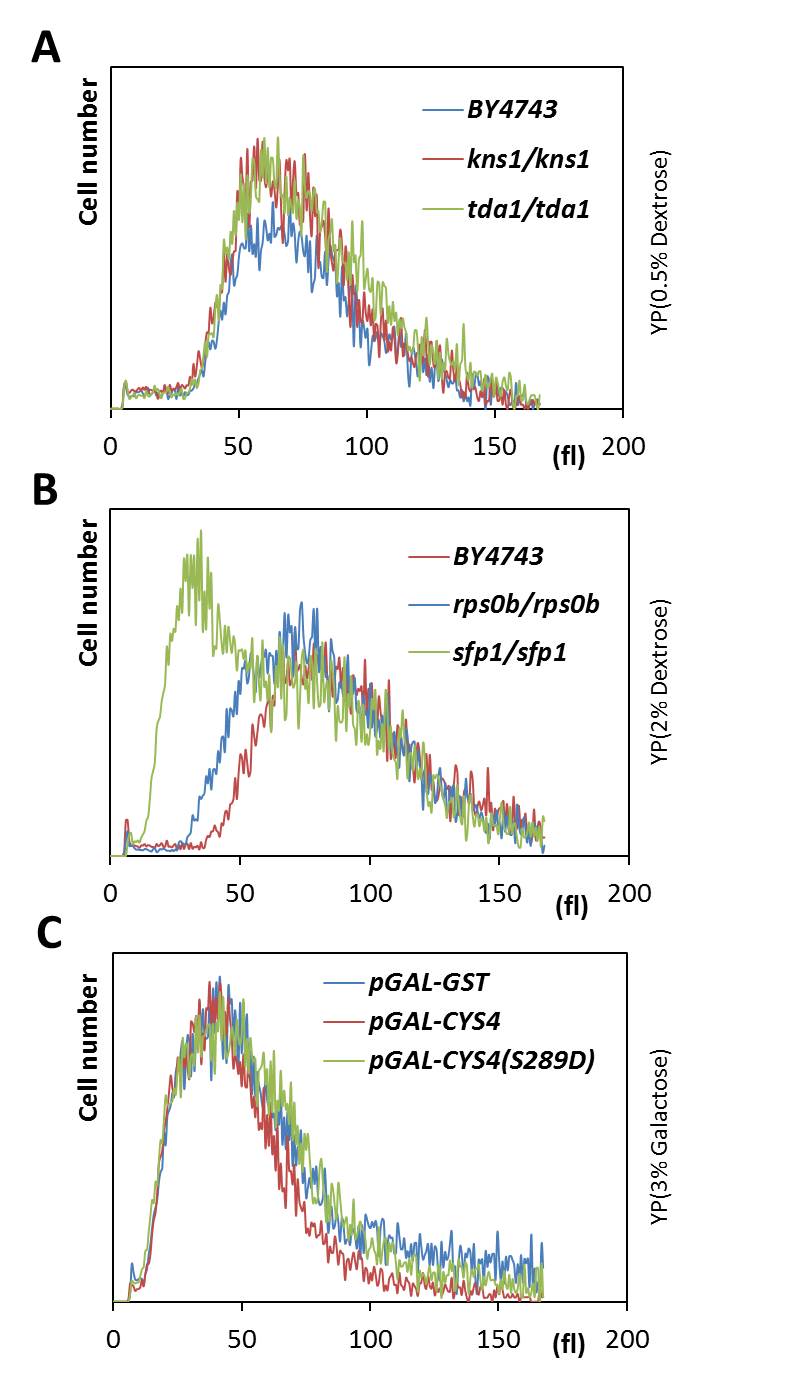

Supplement: Figure S2 — Cell size distributions of asynchronous cultures. The cell size of the indicated cell populations was measured using a channelyzer (see Methods). Cell numbers are plotted on the y-axis and the x-axis indicates size (in fl). A, Size distributions of wild type (BY4743), kns1Δ/kns1Δ and tda1Δ/tda1Δ cells, cultured in YPD (0.5% Dextrose) medium. B, Size distributions of wild type (BY4743), sfp1Δ/sfp1Δ and rps0bΔ/rps0bΔ cells, cultured in YPD (2% Dextrose) medium. C, Size distributions of wild type pGAL-GST, pGAL-CYS4, pGAL-CYS4(S289D) cells, cultured in YPGal (3% Galactose) medium. (JPG) [file pgen.1002590.s003.jpg]

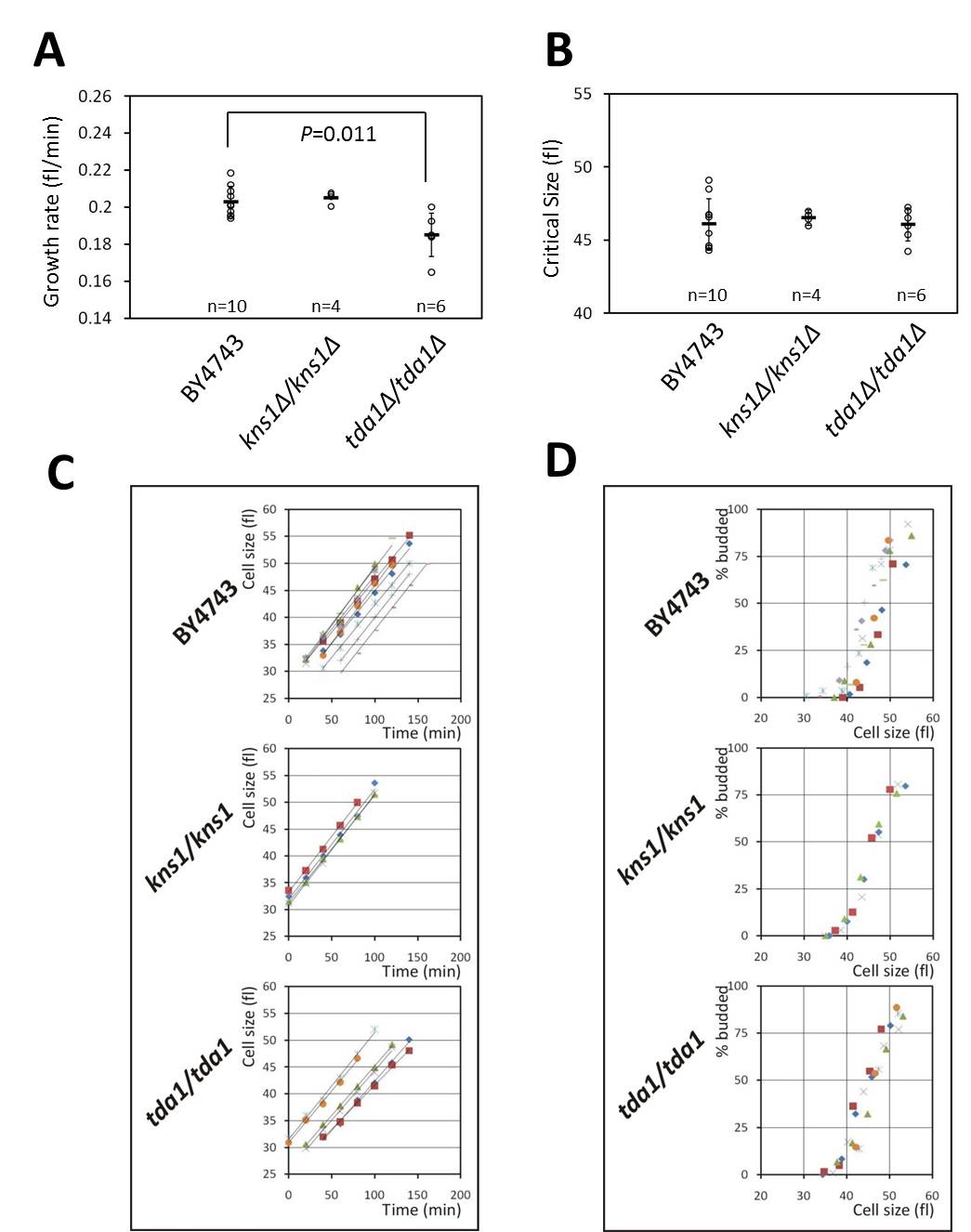

Supplement: Figure S3 — Evaluating false negatives. A, Rate of cell size increase (shown as growth rate, in fl/min) for the indicated strains was measured from synchronous cultures, in rich (YPD-0.5% Dextrose) medium, assuming linear growth. The average value for each strain is shown with a horizontal bar (± sd). B, The critical cell size of the indicated strains (in fl), was measured from the same experiments shown in A. C, Graphs from which we determined the growth rates shown in A. D, Graphs from which we determined the percent of budded cells as a function of cell size, from the same elutriation experiments. The data points shown were from the linear portion of each experiment, when the percentage of budded cells began to increase, and used to determine the critical size for division we show in B. (JPG) [file pgen.1002590.s004.jpg]

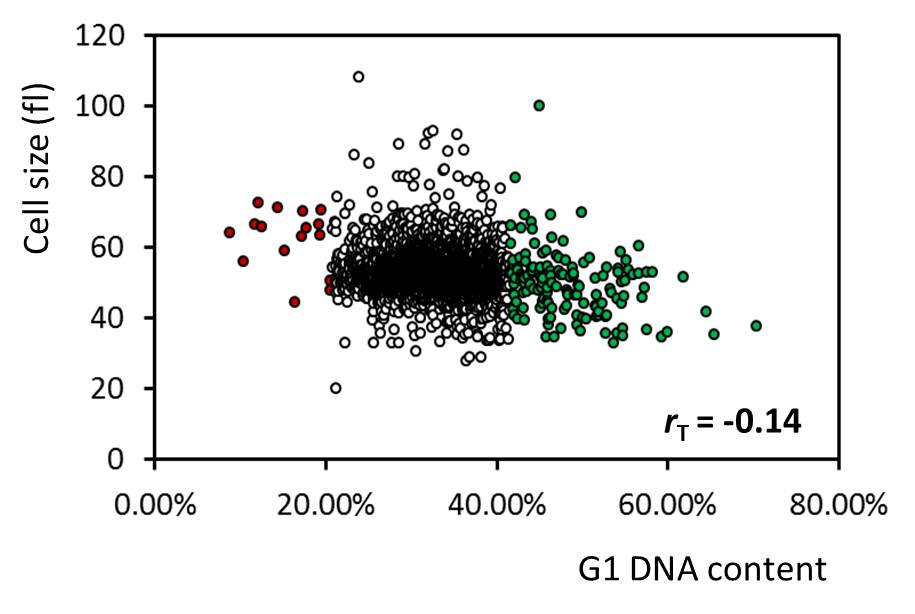

Supplement: Figure S4 — Cell cycle progression correlates weakly with cell size data from stationary phase growth. We plotted the %G1 (x-axis) from all the deletion strains we examined against the diploid median cell size (in fl, y-axis) data of Zhang et al (24), in stationary phase after growth on solid media. We calculated and displayed the r value as in Figure 3. For every gene we included in this analysis, the values we used in this correlation are shown in Dataset S1. (JPG) [file pgen.1002590.s005.jpg]

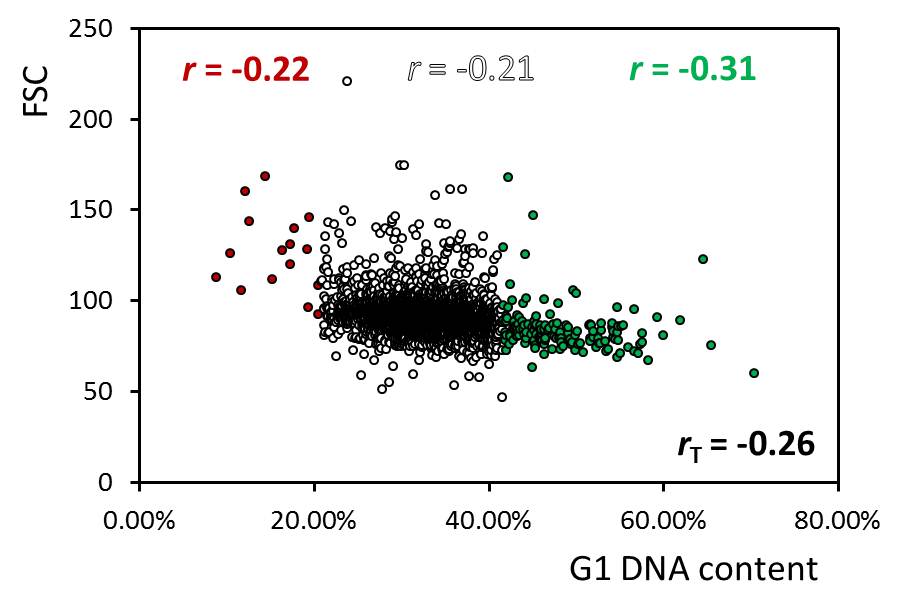

Supplement: Figure S5 — Correlation between DNA content and FSC values. The %G1 is shown on the x-axis, and the forward angle scattering (FSC) values on the y-axis, from all the deletion strains we examined by flow cytometry. We calculated and displayed the r values as in Figure 3. For every gene we included in this analysis, the values we used in this correlation are shown in Dataset S1. (JPG) [file pgen.1002590.s006.jpg]

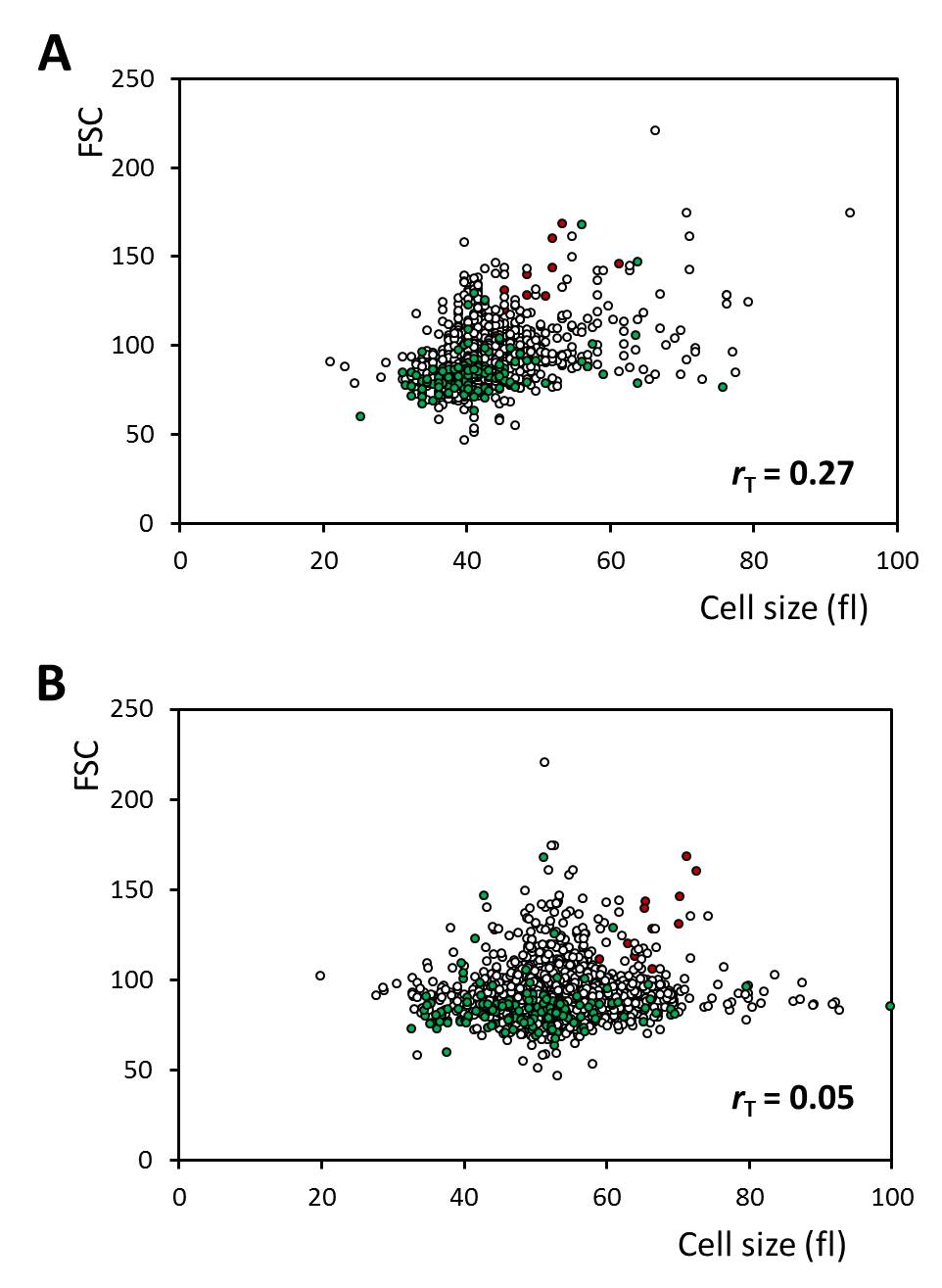

Supplement: Figure S6 — Correlation between FSC and cell size values. We plotted the FSC values (y-axis) from all the deletion strains we examined against the median cell size (in fl, x-axis) data of Jorgensen et al (23) (A), or Zhang et al (24) (B). We calculated and displayed the r values as in Figure 3. For every gene we included in this analysis, the values we used in this correlation are shown in Dataset S1. (JPG) [file pgen.1002590.s007.jpg]

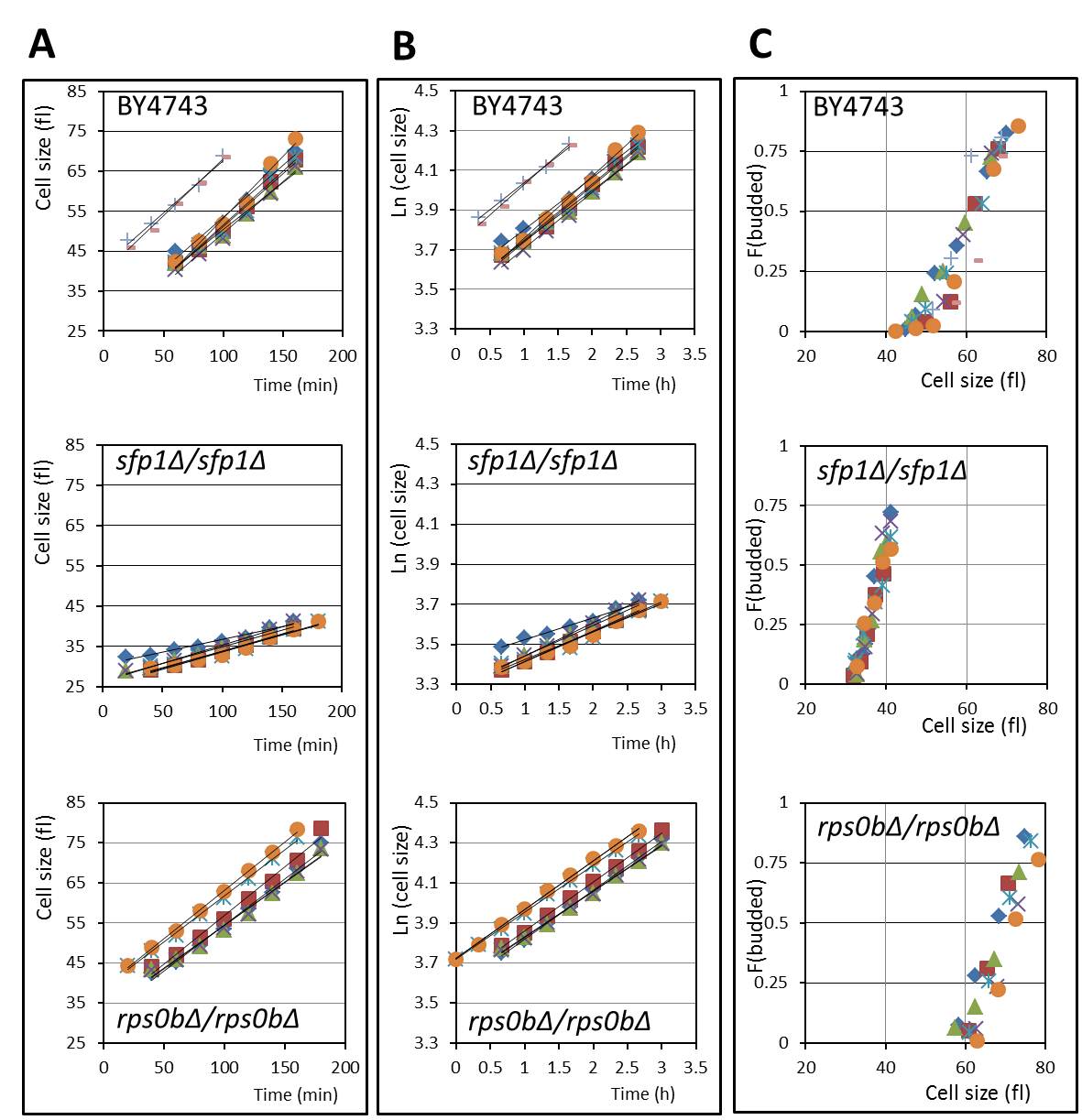

Supplement: Figure S7 — Determining the timing of START in mutants that affect ribosome biogenesis. A, Graphs from which we determined the rate of cell size increase shown in Figure 5A, assuming linear growth. Our measurements were from synchronous cultures, in rich (YPD-2% Dextrose) medium. B, Graphs from which we determined the specific rate of cell size increase constant k, shown in Figure 5B, from the same elutriation experiments shown in A. In this case, we plotted the natural log of the cells size (y-axis), against time (shown in hours, x-axis). C, Graphs of the fraction of budded cells (y-axis) as a function of cell size (in fl, x-axis), from the same elutriation experiments. The data points shown were from the linear portion of each experiment, when the percentage of budded cells began to increase, and used to determine the critical size for division we show in Figure 5C. (JPG) [file pgen.1002590.s008.jpg]

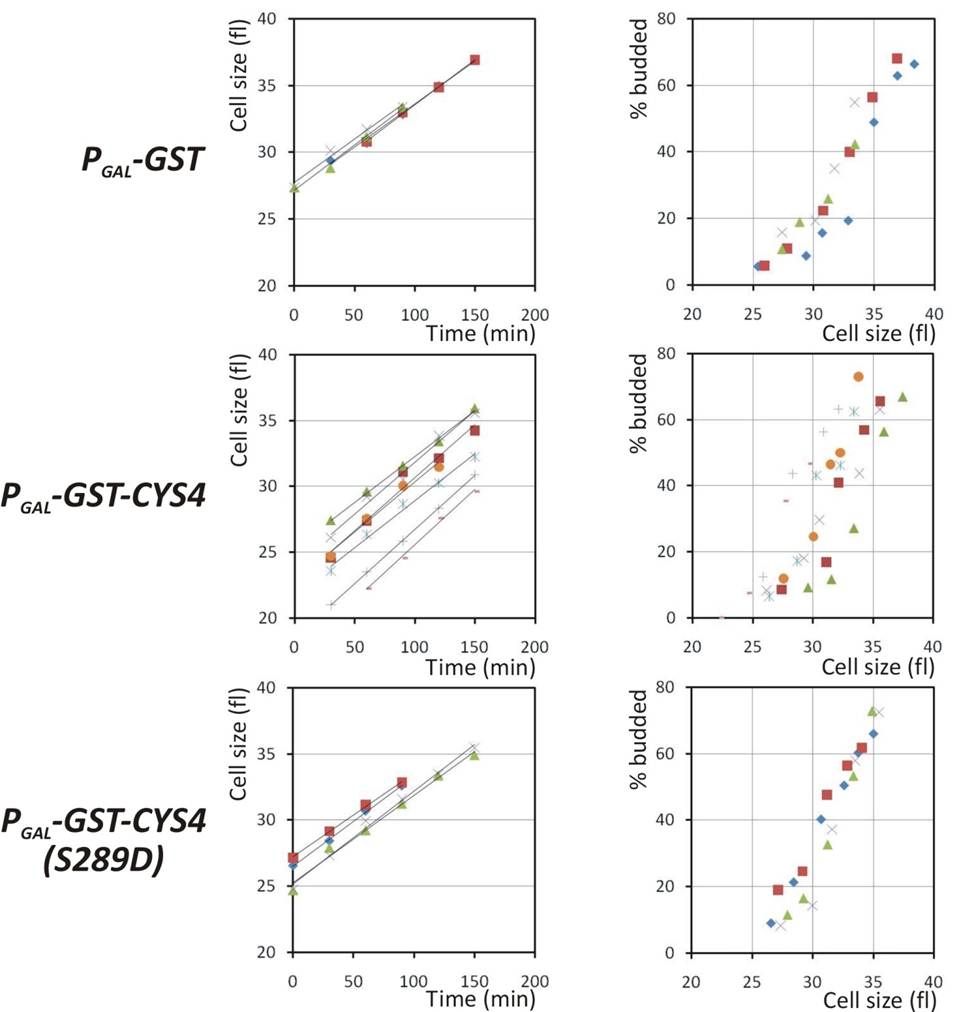

Supplement: Figure S8 — Cell cycle progression of synchronous cultures of PGAL haploid strains, in galactose-containing media. The full data set used to calculate the values shown in Figure 9A and 9B, are shown on the left, and right panels, respectively. Elutriations were done in media that contain galactose and induce expression of the PGAL alleles (see Methods). (JPG) [file pgen.1002590.s009.jpg]

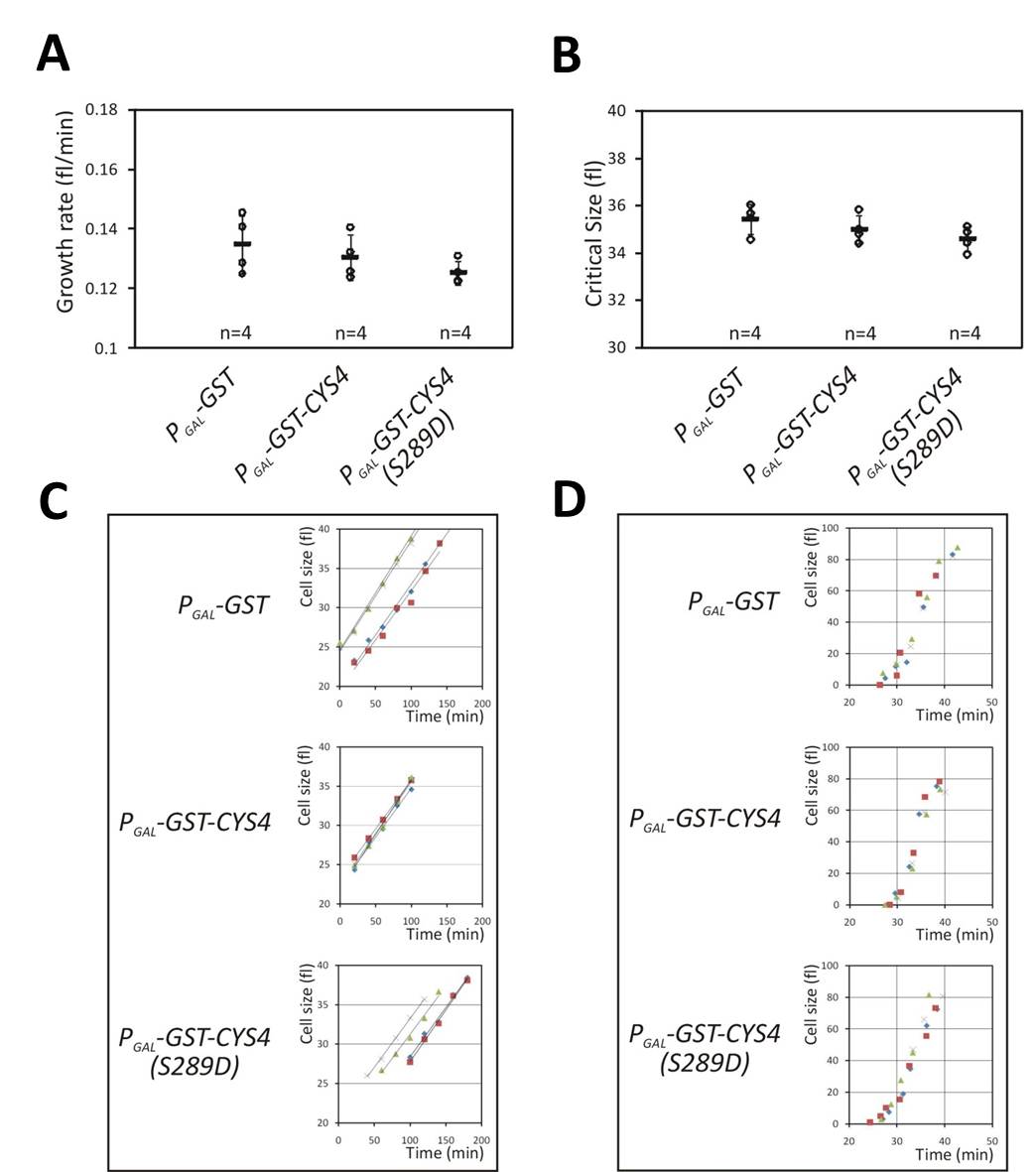

Supplement: Figure S9 — Cell cycle progression of synchronous cultures of PGAL haploid strains, in repressive, glucose-containing media. A, The rate of cell size increase (shown as growth rate, in fl/min) for the indicated strains was measured from synchronous elutriated cultures assuming linear growth, as in Figure 9, in media that contain glucose (YPD-0.5% Dextrose) and repress expression of the PGAL alleles. The average value for each strain is shown with a horizontal bar (± sd). B, The critical cell size of the indicated strains (shown in fl), was measured from the same elutriation experiments shown in A. The rate of cell size increase for each elutriation experiment of the indicated strains is shown on the left panels. C, D, The full data set used to calculate the values shown in A, and B, respectively. (JPG) [file pgen.1002590.s010.jpg]
