# Supplementary material for: A Systematic Analysis of Cell Cycle Regulators in Yeast Reveals That Most Factors Act Independently of Cell Size to Control Initiation of Division
Source: PLoS Genet. 2012 Mar 15;8(3):e1002590. doi: 10.1371/journal.pgen.1002590 (PMC3305459; doi:10.1371/journal.pgen.1002590)
Supplement: Table S1 — S. cerevisiae strains used in this study. (DOCX) [file pgen.1002590.s011.docx]

| **Table S1. *S. cerevisiae* strains used in this study** | | | |
| --- | --- | --- | --- |
| **Strain** | | **Genotype** | **Source** |
| Homozygous diploid  deletions (BY4743  background) | MATa/α *his3Δ1/his3Δ1 leu2Δ0 /leu2Δ0 lys2Δ0/LYS2 MET15/met15Δ0 ura3Δ0/ura3Δ0* *orfΔ::kanMX4/orfΔ::kanMX4* | | Research Genetics-Open Biosystems |
| W303-K699 | MATa *ade2-1 ura3-1 trp1-1 can1-100 leu2-3,112 his3-11,15 GAL psi^+^* | | Bruce Futcher |
| *SCSAH01* | *ura3-1::P_GAL_-GST::URA3 (at URA3)* (W303-K699 otherwise) | | This study |
| *SCSAH02* | *ura3-1::P_GAL_-GST-CYS4::URA3 (at URA3)* (W303-K699 otherwise) | | This study |
| *SCSAH03* | *ura3-1::P_GAL_-GST-CYS4(S289D)::URA3 (at URA3)*  (W303-K699 otherwise) | | This study |
| BY4742 | MATa *his3Δ1* *leu2Δ0* *lys2Δ0* *ura3Δ0* | | Open Biosystems |
| 16696 | *cys4Δ::kanMX4* (BY4742 otherwise) | | Open Biosystems |
| SCMSP178 | *CYS4-13MYC::KanMX4*  (BY4742 otherwise) | | This study |
| SCMSP214 | *CYS4(S289D)-13MYC::KanMX4*  (BY4742 otherwise) | | This study |
