# Supplementary material for: A Systematic Analysis of Cell Cycle Regulators in Yeast Reveals That Most Factors Act Independently of Cell Size to Control Initiation of Division
Source: PLoS Genet. 2012 Mar 15;8(3):e1002590. doi: 10.1371/journal.pgen.1002590 (PMC3305459; doi:10.1371/journal.pgen.1002590)
Supplement: Table S2 — Correspondence between genes that affect cell division when over-expressed, with genes required for normal cell cycle progression. (DOCX) [file pgen.1002590.s012.docx]

**Table S2. Correspondence between genes that affect cell division when over-expressed, with genes required for normal cell cycle progression.**

| **ORF** | **Phenotype** | | **Comment** |
| --- | --- | --- | --- |
|  | Over-expression | Deletion |  |
| YLR052W | G1 |  |  |
| YOR131C | G1 |  |  |
| YHL001W | G1 | ‡NA |  |
| YER028C | G1 |  |  |
| YHR174W | G1 | NA |  |
| YDR117C | G1 |  |  |
| YGR112W | G1 | G1 |  |
| YDR156W | G1 |  |  |
| YCR046C | G1 | †ND |  |
| YDR493W | G1 |  |  |
| YOR065W | G1 |  |  |
| YPR152C | G1 |  |  |
| YDR397C | G1 | NA | essential |
| YIR013C | G1 |  |  |
| YHL031C | G1 | ND |  |
| YPL127C | G1 |  |  |
| YHR070W | G1 | NA | essential |
| YNL167C | G1 | NA |  |
| YMR275C | G1 |  |  |
| **ORF** | **Phenotype** | | **Comment** |
|  | Over-expression | Deletion |  |
| YGL105W | G1 |  |  |
| YLL066W-B | G1 | NA |  |
| YKL052C | G2 | NA | essential |
| YBR131C-A | G2 | NA | Dubious, overlaps YBR131W |
| YOR257W | G2 | NA | essential |
| YCR093W | G2 | NA | essential |
| YGR206W | G2 |  |  |
| YML055W | G2 |  |  |
| YHR172W | G2 | NA | essential |
| YIL138C | G2 |  |  |
| YBL050W | G2 | NA | essential |
| YOR326W | G2 | NA | essential |
| YNL264C | G2 |  |  |
| YDR277C | G2 |  |  |
| YLR123C | G2 |  |  |
| YML052W | G2 |  |  |
| YHR014W | G2 |  |  |
| YHR002W | G2 | NA |  |
| YLR394W | G2 |  |  |
| YJR060W | G2 | ND |  |
| YCL026C-A | G2 |  |  |
| YPR015C | G2 |  |  |
| **ORF** | **Phenotype** | | **Comment** |
|  | Over-expression | Deletion |  |
| YOR286W | G2 | ND |  |
| YGR091W | G2 | NA | essential |
| YDL002C | G2 |  |  |
| YJL077W-A | G2 | NA | Dubious, overlaps YJL077C |
| YML007W | G2 |  |  |
| YER145C | G2 |  |  |
| YLR149C | G2 | ND |  |
| YJL012C | G2 |  |  |
| YLR341W | G2 |  |  |
| YNL188W | G2 | NA | essential |
| YOR195W | G2 |  |  |
| YGR109C | G2 |  |  |
| YBR211C | G2 | NA | essential |
| YDR245W | G2 |  |  |
| YDR033W | G2 |  |  |
| YJL030W | G2 |  |  |
| YDR091C | G2 | NA | essential |
| YIR001C | G2 |  |  |
| YKL078W | G2 | NA | essential |
| YPR190C | G2 | NA | essential |
| YDR266C | G2 |  |  |
| YDL214C | G2 |  |  |
| **ORF** | **Phenotype** | | **Comment** |
|  | Over-expression | Deletion |  |
| YDR001C | G2 |  |  |
| YIR016W | G2 |  |  |
| YBR083W | G2 |  |  |
| YKR067W | G2 |  |  |
| YHR131C | G2 |  |  |
| YFL022C | G2 | NA | essential |
| YDR143C | G2 |  |  |
| YPL174C | G2 | G1 | *NIP100* |
| YOR002W | G2 |  |  |
| YNL283C | G2 |  |  |
| YPL247C | G2 |  |  |
| YJL031C | G2 | NA | essential |
| YPR119W | G2 | G2 | *CLB2* |
| YML053C | G2 |  |  |
| YJL106W | G2 |  |  |
| YMR199W | G2 |  |  |
| YML016C | G2 |  |  |
| YLR189C | G2 |  |  |
| YER007W | G2 |  |  |
| YEL022W | G2 | NA |  |
| YDL192W | G2 |  |  |
| YDR335W | G2 |  |  |
| **ORF** | **Phenotype** | | **Comment** |
|  | Over-expression | Deletion |  |
| YKR029C | G2 |  |  |
| YGR094W | G2 | NA | essential |
| YBL031W | G2 |  |  |
| YGR005C | G2 | NA | essential |
| YGR109W-A | G2 | NA | Ty-transposon |
| YPL116W | G2 |  |  |
| YAR007C | G2 | NA | essential |
| YDL093W | G2 |  |  |
| YOL063C | G2 |  |  |
| YJR125C | G2 |  |  |
| YIL036W | G2 |  |  |
| YOR337W | G2 |  |  |
| YOR007C | G2 |  |  |
| YJL128C | G2 |  |  |
| YIL158W | G2 |  |  |
| YHR177W | G2 |  |  |
| YGL066W | G2 |  |  |
| YMR068W | G2 |  |  |
| YER131W | G2 |  |  |
| YFL037W | G2 | NA | essential |
| YDL155W | G2 |  |  |
| YPR120C | G2 |  |  |
| **ORF** | **Phenotype** | | **Comment** |
|  | Over-expression | Deletion |  |
| YFL039C | G2 | NA | essential |

|  |  |  |  |
| --- | --- | --- | --- |

‡NA, not available. These deletion strains were not in the panel.

†ND, not done. These deletion strains did not pass quality control either when they were generated, or when we analyzed them.
