# Supplementary material for: A Systematic Analysis of Cell Cycle Regulators in Yeast Reveals That Most Factors Act Independently of Cell Size to Control Initiation of Division
Source: PLoS Genet. 2012 Mar 15;8(3):e1002590. doi: 10.1371/journal.pgen.1002590 (PMC3305459; doi:10.1371/journal.pgen.1002590)
Supplement: Table S3 — Correspondence between gene deletions that affect the budding index and the DNA content. (DOCX) [file pgen.1002590.s013.docx]

**Table S3. Correspondence between gene deletions that affect the budding index and the DNA content.**

| **ORF** | **Phenotype** | |
| --- | --- | --- |
|  | BI***** | %G1 |
| YLR226W | Low | ND† |
| YKL068W | Low |  |
| YHL025W | Low | High |
| YOR096W | Low | High |
| YJL089W | Low |  |
| YHR008C | Low | ND |
| YLL007C | Low |  |
| YHL011C | Low | High |
| YKR092C | Low |  |
| YMR060C | Low | High |
| YCL058C | Low | High |
| YOR309C | Low | High |
| YPL257W | Low |  |
| **ORF** | **Phenotype** | |
|  | BI | %G1 |
| YPL240C | Low |  |
| YPL271W | Low | High |
| YPL220W | Low |  |
| YPL171C | Low |  |
| YPL265W | Low |  |
| YPL227C | Low |  |
| YPL193W | Low | High |
| YPL226W | Low | High |
| YPL161C | Low | ND |
| YBR199W | Low |  |
| YBR200W | Low | ND |
| YBR181C | Low | High |
| YPL125W | Low | High |
| YDR140W | Low | High |
| YDR379W | Low |  |
| **ORF** | **Phenotype** | |
|  | BI | %G1 |
| YDR418W | Low | High |
| YDR399W | Low |  |
| YEL001C | Low | ND |
| YEL007W | Low |  |
| YDR378C | Low | High |
| YKL009W | Low | High |
| YKL096W | High |  |
| YKL113C | High |  |
| YKL143W | High |  |
| YKL129C | High |  |
| YOR107W | High |  |
| YKL116C | High |  |
| YKL164C | High |  |
| YKL187C | High |  |
| YOR279C | High |  |
| **ORF** | **Phenotype** | |
|  | BI | %G1 |
| YHR191C | High |  |
| YJL047C | High | Low |
| YGR107W | High |  |
| YPL267W | High |  |
| YPL191C | High |  |
| YPL120W | High |  |
| YPL114W | High |  |
| YPL108W | High |  |
| YBR205W | High |  |
| YBR231C | High |  |
| YDR121W | High | ND |
| YDR122W | High |  |
| YDR055W | High |  |
| YDR073W | High |  |
| YDR135C | High |  |
| **ORF** | **Phenotype** | |
|  | BI | %G1 |
| YDR085C | High |  |
| YDR101C | High |  |
| YDR069C | High |  |
| YDR102C | High |  |
| YDR338C | High |  |
| YDR363W | High |  |
| YDR369C | High |  |
| YDR393W | High |  |
| YDR402C | High |  |
| YEL004W | High |  |
| YCL016C | High |  |
| YKL041W | High |  |
| YKL048C | High |  |
| YPR109W | High |  |
| YPR115W | High |  |
| **ORF** | **Phenotype** | |
|  | BI | %G1 |
| YPR119W | High | Low |
| YPR135W | High | ND |
| YGR188C | High |  |

* BI, Budding Index, as defined in Zettel et al (44).

†ND, not done.
