# Supplementary material for: A Systematic Analysis of Cell Cycle Regulators in Yeast Reveals That Most Factors Act Independently of Cell Size to Control Initiation of Division
Source: PLoS Genet. 2012 Mar 15;8(3):e1002590. doi: 10.1371/journal.pgen.1002590 (PMC3305459; doi:10.1371/journal.pgen.1002590)
Supplement: Table S4 — Gene Ontology enrichment of the “Low G1” group. (DOCX) [file pgen.1002590.s014.docx]

**Table S4. Gene Ontology Enrichment of the “Low G1” group*.**

| **ID** | **Process** | **p-value** |
| --- | --- | --- |
| GO:0022403 | cell cycle phase | 0.0017 |
| GO:0010529 | negative regulation of transposition | 0.0052 |
| GO:0033554 | cellular response to stress | 0.0104 |
| GO:0006974 | response to DNA damage stimulus | 0.0316 |
| GO:0007049 | cell cycle | 0.0426 |
| GO:0000278 | mitotic cell cycle | 0.0438 |
| GO:0000725 | recombination repair | 0.0464 |

***** The analysis was performed with the YeastMine (v. 2011-10-09) feature of the Saccharomyces Genome Database (http://yeastmine.yeastgenome.org/yeastmine).
