# Supplementary material for: A Systematic Analysis of Cell Cycle Regulators in Yeast Reveals That Most Factors Act Independently of Cell Size to Control Initiation of Division
Source: PLoS Genet. 2012 Mar 15;8(3):e1002590. doi: 10.1371/journal.pgen.1002590 (PMC3305459; doi:10.1371/journal.pgen.1002590)
Supplement: Table S5 — Gene Ontology enrichment of the “High G1” group. (DOCX) [file pgen.1002590.s015.docx]

**Table S5. Gene Ontology Enrichment of the “High G1” group*.**

| **ID** | |  | | **Process** | **p-value** | |
| --- | --- | --- | --- | --- | --- | --- |
| GO:0002181 | |  | | cytoplasmic translation | 2.56E^-17^ | |
| GO:0042274 | |  | | ribosomal small subunit biogenesis | 9.35E^-15^ | |
| GO:0042254 | |  | | ribosome biogenesis | 2.24E^-13^ | |
| GO:0022613 | |  | | ribonucleoprotein complex biogenesis | 1.01E^-12^ | |
| GO:0030490 | |  | | maturation of SSU-rRNA | 3.05E^-11^ | |
| GO:0071843 | |  | | cellular component biogenesis at cellular level | 3.58E^-10^ | |
| GO:0000462 | |  | | maturation of SSU-rRNA from tricistronic rRNA transcript (SSU-rRNA, 5.8S rRNA, LSU-rRNA) | 1.15E^-09^ | |
| GO:0044085 | |  | | cellular component biogenesis | 1.10E^-07^ | |
| GO:0006364 | |  | | rRNA processing | 1.68E^-06^ | |
| GO:0016072 | |  | | rRNA metabolic process | 3.71E^-06^ | |
| GO:0006412 | |  | | translation | 3.81E^-06^ | |
| GO:0042255 | |  | | ribosome assembly | 3.07E^-05^ | |
| GO:0034470 | |  | | ncRNA processing | 5.04E^-05^ | |
| GO:0000028 | |  | | ribosomal small subunit assembly | 1.13E^-04^ | |
| GO:0070925 | |  | | organelle assembly | 1.59E^-04^ | |
| GO:0006396 | |  | | RNA processing | 2.57E^-04^ | |
| GO:0034660 | |  | | ncRNA metabolic process | 8.03E^-04^ | |
| GO:0071841 | |  | | cellular component organization or biogenesis at cellular level | 0.0017 | |
|  | |  | |  |  | |
| **ID** | |  | | **Process** | **p-value** | |
| GO:0022618 | |  | | ribonucleoprotein complex assembly | 0.0035 | |
| GO:0007035 | |  | | vacuolar acidification | 0.0053 | |
| GO:0045851 | |  | | pH reduction | 0.0053 | |
| GO:0051452 | |  | | intracellular pH reduction | 0.0053 | |
| GO:0071826 | |  | | ribonucleoprotein complex subunit organization | 0.0056 | |
| GO:0006407 | |  | | rRNA export from nucleus | 0.0070 | |
| GO:0030641 | |  | | regulation of cellular pH | 0.0070 | |
| GO:0051029 | |  | | rRNA transport | 0.0070 | |
| GO:0051453 | | | regulation of intracellular pH | | 0.0070 |  |
| GO:0071840 | | cellular component organization or biogenesis | | | 0.0113 |  |
| GO:0006885 | | regulation of pH | | | 0.0193 |  |
| GO:0000478 | | endonucleolytic cleavage involved in rRNA processing | | | 0.0270 |  |
| GO:0000479 | | endonucleolytic cleavage of tricistronic rRNA transcript (SSU-rRNA, 5.8S rRNA, LSU-rRNA) | | | 0.0270 |  |
| GO:0010467 | | gene expression | | | 0.0407 |  |
| GO:0015931 | | nucleobase, nucleoside, nucleotide and nucleic acid transport | | | 0.0417 |  |
| GO:0050658 | | RNA transport | | | 0.0495 |  |
| GO:0051236 | | establishment of RNA localization | | | 0.0495 |  |

***** The analysis was performed with the YeastMine (v. 2011-10-09) feature of the Saccharomyces Genome Database (http://yeastmine.yeastgenome.org/yeastmine).
